# An Orbitrap/Time-of-Flight Mass Spectrometer for Photofragment Ion Imaging and High-Resolution Mass Analysis of Native Macromolecular Assemblies

**DOI:** 10.1021/jasms.3c00053

**Published:** 2023-06-15

**Authors:** Anjusha Mathew, Frans Giskes, Alexandros Lekkas, Jean-François Greisch, Gert B. Eijkel, Ian G. M. Anthony, Kyle Fort, Albert J. R. Heck, Dimitris Papanastasiou, Alexander A. Makarov, Shane R. Ellis, Ron M. A. Heeren

**Affiliations:** † Maastricht MultiModal Molecular Imaging (M4i) Institute, Division of Imaging Mass Spectrometry (IMS), Maastricht University, 6229 ER Maastricht, The Netherlands; ‡ Fasmatech Science and Technology, Demokritos NCSR, 15310 Agia Paraskevi, Athens, Greece; § Biomolecular Mass Spectrometry and Proteomics, Bijvoet Centre for Biomolecular Research and Utrecht Institute for Pharmaceutical Sciences, Utrecht University, Padualaan 8, 3584 CH Utrecht, The Netherlands; ∥ Netherlands Proteomics Center, Padualaan 8, 3584 CH Utrecht, The Netherlands; ⊥ Thermo Fisher Scientific (Bremen) GmbH, 28199 Bremen, Germany; # Molecular Horizons and School of Chemistry and Molecular Bioscience, University of Wollongong, Wollongong, New South Wales 2522, Australia

**Keywords:** macromolecular assemblies, Orbitrap mass spectrometry, orthogonal time-of-flight mass spectrometry, photofragment ion imaging, Timepix detector, UV photodissociation

## Abstract

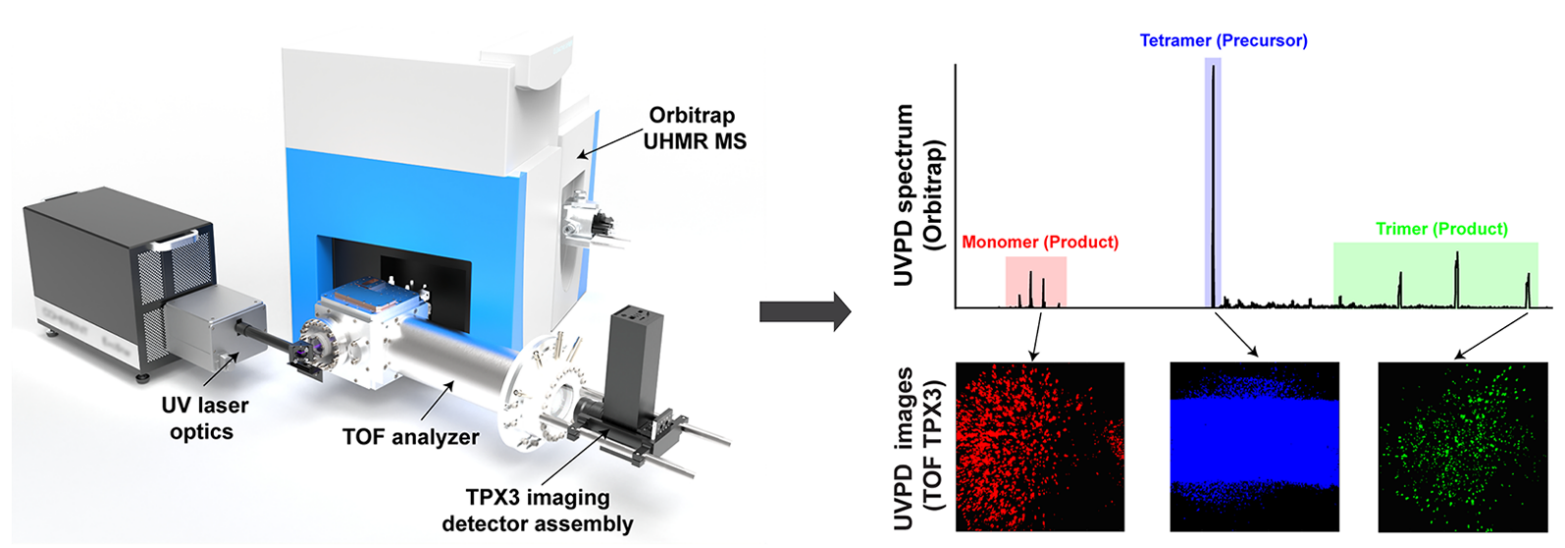

We discuss the design, development, and evaluation of an Orbitrap/time-of-flight (TOF) mass spectrometry (MS)-based instrument with integrated UV photodissociation (UVPD) and time/mass-to-charge ratio (*m*/*z*)-resolved imaging for the comprehensive study of the higher-order molecular structure of macromolecular assemblies (MMAs). A bespoke TOF analyzer has been coupled to the higher-energy collisional dissociation cell of an ultrahigh mass range hybrid quadrupole-Orbitrap MS. A 193 nm excimer laser was employed to photofragment MMA ions. A combination of microchannel plates (MCPs)-Timepix (TPX) quad and MCPs-phosphor screen-TPX3CAM assemblies have been used as axial and orthogonal imaging detectors, respectively. The instrument can operate in four different modes, where the UVPD-generated fragment ions from the native MMA ions can be measured with high-mass resolution or imaged in a mass-resolved manner to reveal the relative positions of the UVPD fragments postdissociation. This information is intended to be utilized for retrieving higher-order molecular structural details that include the conformation, subunit stoichiometry, and molecular interactions as well as to understand the dissociation dynamics of the MMAs in the gas phase.

## Introduction

Mass spectrometry (MS) has emerged as a versatile and powerful tool to study molecular structural features of macromolecular assemblies (MMAs).^1−4^ The MMAs are a broad range of important large (molecular weight range: kDa to MDa) complex biological ensembles of proteins, nucleic acids, carbohydrates, lipids, metabolites, metal ions, ligands, etc. The majority of the previous studies on MMAs targeted protein complexes or the complexes formed by binding of cofactors such as lipids, DNA or RNA, ligands, and metal ions to the proteins, termed multiproteoform complexes (MPCs).^5−10^ Here, we exclusively focus on the techniques for the molecular structural elucidation of MPCs.

With the advancements in MS instrumentation, mass spectrometers that provide ultrahigh mass resolution (>10^6^ at *m*/*z* (mass-to-charge ratio) = 200), ppb to subppm mass accuracy, wide and high *m*/*z* range, and femtomole to attomole detection sensitivity are now available.^11−20^ Often, a combination of liquid chromatography (LC) coupled online to a nanoelectrospray ionization (nESI) source and mass spectrometers with the above-mentioned features and MS/MS capabilities are used for the characterization of the protein sequence. Several MS-based approaches such as native MS (nMS), ion mobility MS (IM MS), affinity purification MS (AP MS), hydrogen–deuterium exchange MS (HDX MS), cross-linking MS (XL MS), and other MS-based footprinting techniques have proven to be complementary to structural biology tools such as cryogenic-electron microscopy, nuclear magnetic resonance, and X-ray crystallography.^1−3,21−29^ These MS-based tools are capable of retrieving several higher-order structural features like subunit stoichiometry and interaction sites of MPCs along with the high-resolution molecular information. These methods are often coupled together and/or integrated with various ion fragmentation methods such as collision-induced dissociation (CID)/higher-energy collisional dissociation (HCD), electron-capture dissociation (ECD), electron-transfer dissociation (ETD), surface-induced dissociation (SID), ultraviolet photodissociation (UVPD), infrared multiphoton dissociation (IRMPD), EThCD (ETD supplemented with HCD), activated ion ETD (AI-ETD, IRMPD followed by ETD), etc. to obtain structural information.^30−38^ However, little information on the 3D conformation of the MMA has been provided with any of these MS-based techniques.

Here, we discuss the design and development of an innovative MS-based instrument that targets comprehensive molecular and structural analysis of MMAs at the same time using only picomoles of sample from solution.^39^ A combination of a Thermo Scientific Q Exactive ultrahigh mass range (UHMR) hybrid quadrupole-Orbitrap mass spectrometer^40−44^ and a custom-designed orthogonal time-of-flight (TOF) mass analyzer with an integrated 193 nm excimer laser^40,45−47^ and two position-and-time sensitive Timepix (TPX) detectors^48−53^ was used for the initial characterization of the system presented here. The Orbitrap/TOF system allows the *m*/*z*-resolved imaging of UV-generated products from the precursor MMA ions, which can be utilized to understand the energetics of the MMA dissociation process as the TOF analyzer can retain the relative positions of the product ions following the fragmentation process until they reach the TPX imaging detector. Moreover, the instrument is capable of sending the UV-generated product ions back to the Orbitrap mass analyzer to obtain the high-resolution UVPD mass spectrum that can provide several higher-order structural details of MMAs including conformation, subunit stoichiometry, and molecular interactions.

The work described in this paper focuses on the ion optics design, development, and evaluation of the Orbitrap/TOF system with integrated pixelated TPX detectors and UVPD. A description of the instrument design, system configurations, modes of operation, and associated ion optics simulations of the custom-designed TOF analyzer are provided in the first section. This is followed by a comprehensive characterization of the system divided into three sections: characterization of the Orbitrap/TOF system with (i) axially and orthogonally coupled discrete-dynode electron multiplier (EM) detectors (ii) axial microchannel plates (MCPs)-TPX quad and orthogonal MCP-phosphor screen (P47)-TPX3CAM imaging detector assemblies, and (iii) coaxial 193 nm excimer laser optics and an orthogonal MCP-P47-TPX3CAM imaging detector assembly.

## Materials and Methods

### Materials

Ubiquitin (8.6 kDa) from bovine erythrocytes, concanavalin A (102 kDa) from *Canavalia ensiformis*, and ammonium acetate were all purchased from Sigma-Aldrich (Zwijndrecht, The Netherlands). Cesium iodide (CsI; 392.7 to 11 304 Da) was obtained from Thermo Fisher Scientific, The Netherlands. Methanol, isopropanol, and LC–MS grade water were purchased from Biosolve (Valkenswaard, The Netherlands).

### Sample Preparation

Ubiquitin was dissolved in 1:1 methanol:water (v:v) to a concentration of 5 μM. Concanavalin A was first dissolved to a stock concentration of 100 μM in LC–MS grade water and then buffer exchanged with 200 mM ammonium acetate at pH 6.8 using a 30 kDa molecular weight cutoff (MWCO) Amicon Ultra centrifugal filter (Millipore, Merck KGaA, Germany) to a final monomer concentration of 5 μM. CsI was prepared as a 2 mg/mL solution in 1:1 isopropanol:water (v:v).

### Instrumentation

**Figure 1:**
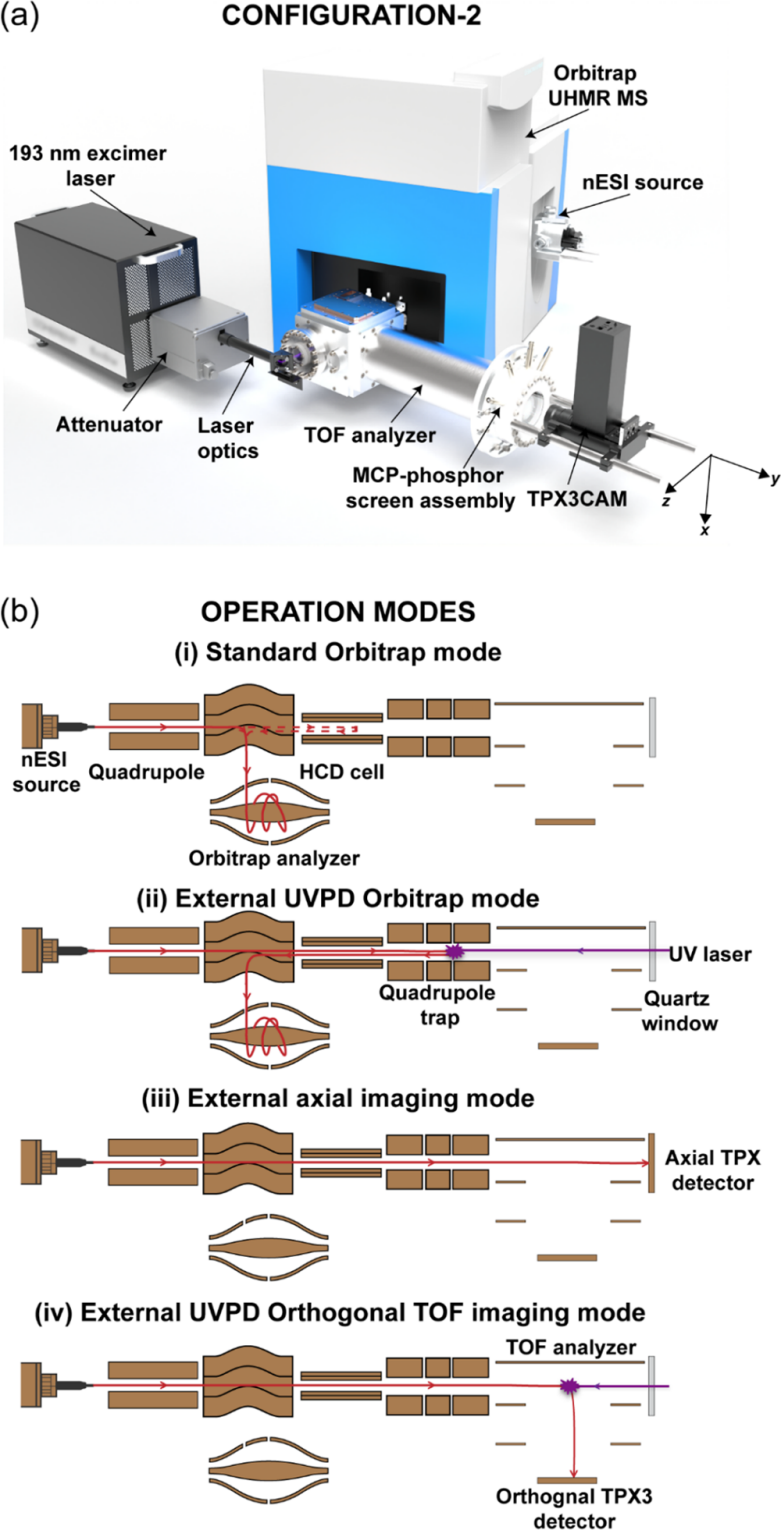
(a) Schematic (not to scale) of the Orbitrap/TOF instrument with coaxial 193 nm excimer laser optics and an orthogonal MCP-P47-TPX3CAM imaging detector assembly (System configuration 2). (b) Different operation modes of the instrument. The trajectories of the precursor/product ions and laser beam are shown in red and violet, respectively.

**Figure 2:**
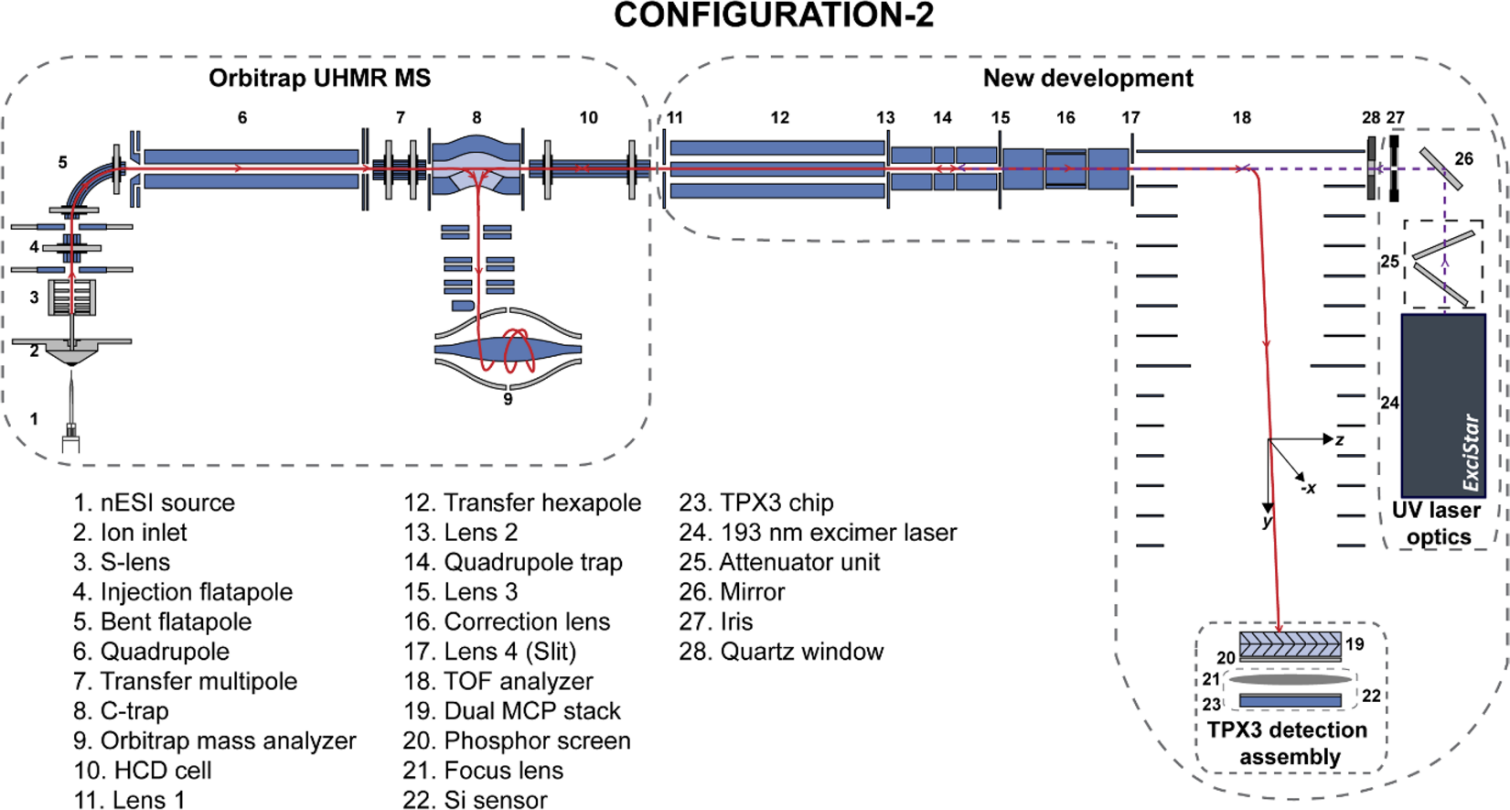
Detailed ion and laser optics schematic (not to scale) of Configuration 2 of the Orbitrap/TOF system.

The comprehensive characterization of the Orbitrap/TOF system was performed in three different configurations using (i) two axially and orthogonally coupled discrete-dynode EM detectors (Configuration 1a), (ii) axial MCP-TPX quad and orthogonal MCP-phosphor screen (P47)-TPX3CAM imaging detector assemblies and orthogonal 193 nm excimer laser optics (Configuration 1b, Figures S1–S2), and (iii) coaxial 193 nm excimer laser optics and an orthogonal MCP-P47-TPX3CAM imaging detector assembly (Configuration 2, Figures 1a and 2). Note that System configurations 1a and b have solely been employed for testing purposes. All the UVPD experiments shown in this Article were conducted in the System configuration 2.

#### Orbitrap/TOF MS

A commercial Q Exactive UHMR hybrid quadrupole-Orbitrap mass spectrometer (Thermo Fisher Scientific, Bremen, Germany) was coupled with a custom-designed orthogonal TOF analyzer, a 193 nm ExciStar XS 200 series excimer laser (Coherent Laser Systems GmbH & Co. KG, Göttingen, Germany), two nonimaging discrete-dynode EM detectors, and two imaging detectors: MCP-TPX quad and MCP-P47-TPX3CAM. The electrometer located at the rear of the HCD cell of the Orbitrap MS was removed, and a transfer hexapole (Element 12, Figure 2) was installed to control ion transport into the TOF region. The ion cloud is collisionally cooled in a linear quadrupole trap (LQ-trap, Element 14, Figure 2) that is positioned in between the hexapole and TOF analyzer (Element 18, Figure 2), and it can be further conditioned by the correction lens (Element 16, Figure 2) as it travels to the TOF region. The ions are directed either toward the axial detection assembly (Configuration 1a and b) or to the orthogonal detection assembly through the TOF analyzer. The details regarding the geometry and ion optical design of the TOF analyzer will be discussed in the next section.

#### Laser Optics

A parallel coherent beam of 193 nm UV photons with a maximum repetition rate of 200 Hz was generated with an ExciStar XS 200 series excimer laser filled with an argon fluoride gas mixture (Element 24, Figure 2). The laser produces 7 ns rectangular pulses of dimensions 6 × 2.5 mm (VxH), with functional energies ranging from 0.5 to 5 mJ/pulse. The UV laser beam was guided to the mass spectrometer through a quartz window via a periscope assembly, equipped with 45°, 193 nm mirrors (Laseroptik GmbH, Garbsen, Germany) mounted on micropositioners and ring-actuated iris diaphragms (Thorlabs, Newton, USA). Two UVPD configurations were evaluated. In Configuration 1b (Figures S1–S2), the laser optics (Elements 24–27, Figure S2) have been installed in such a way that the laser beam (*y*) can interact with the ion cloud (*z*) orthogonally at any segment of the LQ-trap or correction lens. However, even at high laser energies (>3.5 mJ at the laser device exit) and high repetition rates (>175 Hz), the fragmentation efficiency was extremely low due to the nonoptimal overlap of the orthogonal laser beam with the ion cloud. The experimental setup has been modified in Configuration 2 (Figures 1a and 2) by replacing the axial TPX quad assembly with a quartz window (Element 28, Figure 2), which allows the UV laser beam to interact coaxially with the ion cloud that resulted in a higher fragmentation efficiency compared to the Configuration 1b. All the UVPD experiments shown in this Article are conducted in the System configuration 2. A manually operated attenuator module (Coherent Laser Systems GmbH & Co. KG, Göttingen, Germany) was installed in front of the 193 nm laser for the fine control of the laser energy in Configuration 2 (Element 25, Figure 2). The HCD exit test point signal was used to generate TTL trigger pulses with a digital pulse generator (DG535, Stanford Research Systems, Sunnyvale, USA) for the laser.

#### Discrete-Dynode EM Detection Systems

Two discrete-dynode EMs (ETP Electron Multipliers, Clyde, Australia) with dimensions of 10 × 14 and 8 × 12 mm were coupled axially and orthogonally through the TOF analyzer, respectively, for the initial characterization of the custom designed part (Configuration 1a). The discrete-dynode EM signals were extracted using a fast oscilloscope (∼500 MHz and 4 GS/s, LeCroy LT372). The signal from the HCD exit test point was used to trigger the oscilloscope. The data acquired using the oscilloscope is saved as text files using the Scope Explorer software (LeCroy Corporation, New York, USA).

#### TPX Detection Systems

The discrete-dynode EMs were replaced after the initial characterization by time-and position-sensitive charge detectors from the Timepix (TPX) family (Medipix consortium, CERN, Geneva, Switzerland). The axial imaging detection assembly consists of a dual MCP chevron stack–TPX^54^ quad system mounted in vacuum (Configuration 1b). This system has previously been coupled to MALDI (matrix-assisted laser desorption/ionization)-axial TOF Bruker Ultraflex III MS.^48,49^ In this study, TPX has been operated in time-of-arrival (TOA) mode, in which the time of activation of each pixel is measured along with pixel coordinates with respect to an external trigger. The TPX data is read out via a ReLAXD (high-Resolution Large-Area X-ray Detector) readout board with a speed of 1 Gbit/s.^55^ The TPX was triggered at a rate of 1–2 Hz using the HCD exit lens trigger pulse via a digital pulse and delay generator (DG535, Stanford Research Systems). The axial TPX data were recorded using a 100 ns TPX clock width, corresponding to a maximum measurement window of 1181 μs for each measurement cycle.

The imaging assembly mounted on the orthogonal TOF MS consists of an MCP-P47-TPX3CAM detector system (Configurations 1b and 2) that was previously employed in Ultraflex III MS.^53^ Briefly, each ion impact on the MCP-P47-TPX3CAM detection assembly leads to a cascade of secondary electrons within the MCP that is subsequently converted to photons by the scintillator (P47). The photons create local electron–hole pairs in the Si-coated TPX3^56^ within the TPX3CAM that results in a detectable current on individual pixels of the TPX3 chip. The TPX3 chip consists of a 256 × 256 pixel matrix with a pixel pitch of 55 μm. In contrast to its predecessor TPX chip, where the readout is frame-based, the readout from TPX3 is event-based, and event data is immediately sent out upon the activation of each pixel. If a signal causes a crossing of the energy threshold, then the hit is registered along with the pixel coordinates, TOA, and time taken for the signal to fall below the threshold, which is referred to as the time-over-threshold (TOT). The dead time of individual pixels to process and store the information after they were hit is about 475 ns plus the corresponding TOT. The TPX3 data is acquired by Speedy PIxel Detector Readout (SPIDR) system (Nikhef, Amsterdam, The Netherlands) and transferred to the acquisition computer with a speed of 1 Gbit·s^–1^.^57^ The SPIDR has an internal time-to-digital converter (TDC), which is able to time stamp incoming digital pulses with 260 ps precision synchronously with the TPX3 hits. This feature is needed to provide an external time reference. The TPX3 and internal TDC of the SPIDR were triggered at a rate of 1–2 Hz using the HCD exit trigger pulse via a digital pulse and delay generator (DG535, Stanford Research Systems). The TPX3 data was recorded at a time resolution of 1.5625 ns with a maximum measurement window of 180 μs for each measurement cycle.

The external high-voltage power supplies from Applied Kilovolt (West Sussex, UK), AMOLF (Amsterdam, The Netherlands), and FuG Elektronik GmbH (Schechen, Germany) were used to power the discrete-dynode EMs, MCPs, and phosphor voltages, respectively. The pulse generator and oscilloscope settings were set manually. All other parameters are controlled by the Q Exactive UHMR tune software (version 2.11 build 3005, Thermo Fisher Scientific, Bremen, Germany) and custom-developed instrument control software (Fasmatech, Athens, Greece). The data acquisition parameters and event sequences used for the generation of each figure are shown in Tables S1–S4 and Figures S14–18, S22, and S25–26.

### Data Analysis

The SoPhy (Software for Physics, Amsterdam Scientific Instruments, Amsterdam, The Netherlands) software package versions 1.5.7 and 1.6.3 were used for the TPX and TPX3 chip’s control and data acquisition, respectively. A total of a hundred measurement cycles (frames) were collected and summed for each TPX/TPX3 data set. The raw files were subsequently analyzed using in-house developed software written in MATLAB (R2018a, MathWorks Inc., Natick, MA, USA).

### Ion Optics Simulations

SIMION 8.1 (Scientific Instrument Services, Ringoes, USA) and SIMAX (MSCUBE, Ponsonby, New Zealand) software packages were used for the ion optics design of the custom-built TOF analyzer. The 3D potential arrays (.pa files) of the TOF analyzer and associated ion optics of the custom-designed part built and refined using the SIMION were exported to the SIMAX software. The time-dependent voltage signals and isotropically distributed ion groups (.ic8 files, without initial axial velocity (*v*_*z*_)) were defined using the SIMAX GUI. *v*_*z*_ component was added to the .ic8 files in Excel, and the updated.ic8 files were then reloaded to the SIMAX. All ion optical simulations were performed using SIMAX.

## Results and Discussion

### Modes of Operation and TOF Analyzer Design

The new instrument consists of two mass analyzers: a modified commercial high-resolution Orbitrap MS and a newly developed orthogonal TOF system. The instrument is equipped with a static nESI source at the entrance of the Orbitrap MS (Element 1, Figure 2) for the ionization of the MMAs in their pseudonative state, by maintaining the noncovalent interactions. This versatile system allows different modes of operation using advanced ion optics for ion manipulation and steering. These operational modes are (1) standard Orbitrap MS acquisition; (2) external Orbitrap MS acquisition with or without (w/wo) UVPD; (3) external axial imaging, and (4) external orthogonal TOF MS imaging w/wo UVPD (Figure 1b). Each of these modes can take advantage of the quadrupole mass filter (Element 6, Figure 2) within the Orbitrap MS to select a specific *m*/*z* of interest. Selected ions can be directed toward the LQ-trap (Element 14, Figure 2) of the custom-designed system, where they are stored for later usage. The different operational modes will be elaborated in the following paragraphs.

#### Standard Orbitrap Mode

The ability to maintain normal Orbitrap UHMR MS operation was an essential design criterion. As a result, the new LQ-trap-TOF analyzer addition only replaces the HCD cell electrometer and does not interfere with normal operation.

#### External UVPD Orbitrap Mode

A hexapole ion guide (Element 12, Figure 2) transfers the ions from the Orbitrap MS through the HCD cell to the segmented LQ-trap of the new (external) instrument (Elements 11–18, Figure 2). In external UVPD Orbitrap mode, the UV laser beam interacts with a large number of precursor ions at the LQ-trap. The precursor ions are radially and axially confined to a well-focused ion cloud by the collision with argon gas in the LQ-trap prior to UVPD to ensure maximum ion–photon interaction. The UV-generated fragments are sent back to the Orbitrap analyzer (Element 9, Figure 2) to obtain a high-resolution UVPD mass spectrum. This mode is employed for the retrieval of several higher-order structural features of MMAs including proteoform composition, subunit stoichiometry, and interactions.

#### External Axial Imaging Mode

In Configuration 1b (Figures S1–S2), the system can be operated in the external axial imaging mode, in which the MMA ions stored in LQ-trap are directed to the axial MCP-TPX quad detection assembly for imaging. The TPX registers both the arrival time and arrival position of the ion cloud. This mode is extremely suitable for the temporal and spatial analysis of the ion package emitted from the LQ-trap. Note that the TOF spectrum acquired on the TPX quad is of poor quality due to the absence of a strong acceleration field and a short flight path from the LQ-trap to the axial detector.

#### External UVPD Orthogonal TOF Imaging Mode

The main purpose of this instrument is to determine the spatial and temporal distribution of the MMA’s fragments using a TOF MS imaging approach. This is achieved in the external UVPD orthogonal TOF imaging mode. The precursor MMA ions stored in the LQ-trap are sent toward the orthogonal TOF analyzer; on the way, they interact with photons in one of the locations between the LQ-trap and TOF analyzer (Elements 14–18, Figure 2). The TOF-separated UV-generated MMA fragments are then accelerated toward the MCP-P47-TPX3CAM imaging assembly for time (*m*/*z*)-resolved imaging. The ion optical design of the TOF analyzer ensures that the relative positions of the fragments are maintained as they separate from each other following the dissociation process until they reach the TPX3 detector assembly. The TPX3 registers both the arrival time and arrival position of each subunit released from the MMA ions. The arrival time information can be used for the generation of the mass spectrum. The arrival coordinates provide the spatial distribution of the product ions at the detection assembly, which is hypothesized to reflect their relative positions and trajectories following the fragmentation process. This can be used to retrieve a large amount of critical information related to the translational energetics of the fragmentation process of the MMAs.^58^

#### TOF Analyzer Design

The TOF analyzer design criteria encompass the following: (i) sufficient time resolution to distinguish complementary subunit fragments generated from MMA ions, (ii) maintaining the relative positions of subunit fragments formed from a single MMA ion, following the dissociation process until they reach the orthogonal TPX3CAM detection assembly, and (iii) ensure that all ions, regardless of angular divergence hit within a maximum 40 mm diameter area of the MCP detector. A TOF analyzer with a two-stage acceleration field (s and d fields) and a bias electrode was designed to meet the aforementioned requirements. The voltage division and dimensions of each electrode of the TOF analyzer are shown in Figure S3. In the design phase, the whole system and its behavior was modeled using SIMION and SIMAX. The Supporting Information describes in detail the ion optical design and simulations of the TOF analyzer (Section “Ion Optical Design of TOF Analyzer” and Figures S4–S12). Briefly, the expected fragments from two commonly used proteins in MS, ubiquitin (∼8.6 kDa) and dimeric concanavalin A (∼51 kDa) were sent to the orthogonal detector to simulate the performance of the TOF analyzer. The simulated fragment ion trajectories, detector images, and TOF spectra were examined under various ion optics settings. The simulation results suggest that the optimum conditions for the operation of the instrument in the external UVPD orthogonal TOF imaging mode are the following: ratio of V_s_ to V_d_ ≥ 0.5, distance from the pusher to the detector = 655 mm, fragmentation location at the center of the pusher, and detector required to be off-centered in the *z*-direction by 20 to 40 mm).

### Characterization of the Orbitrap/TOF Instrument with Imaging Detectors

The spectral, nonimaging performance of the instrument was evaluated under normal operational conditions in the System configuration 1a (see Supporting Information, Section “Characterization of the Orbitrap/TOF Instrument without Imaging Detectors and UV Laser”, Tables S1–S2, and Figures S13–22). Subsequently, the influence of the custom-built TOF analyzer and the associated ion optics on the ion trajectories was investigated by the visualization of the spatial distribution of the ion cloud with axial and orthogonal imaging detectors. The MCP-TPX quad^48−50,52,59−67^ and MCP-P47-TPX3CAM^51,53,68−77^ assemblies coupled with the Orbitrap/TOF instrument are deployed for this purpose (Configuration 1b, Figures S1–S2). Singly charged CsI ions of mass 5589 Da were selected using the quadrupole mass filter. The spatial distribution of these selected ions was studied with the orthogonal TPX3CAM and axial TPX quad assemblies by operating the instrument in external orthogonal TOF imaging and external axial imaging modes, respectively.

The ions are initially accelerated in the *z*-direction as they leave the HCD cell of the Orbitrap MS and travel toward the TOF region/axial detector (Figure 2). Ions are imaged in the *xy* plane when operated in the external axial imaging mode. In external orthogonal TOF imaging mode, the pusher pulses ions in the *y*-direction for the TOF separation and orthogonal spatial profile measurements and are detected by the TPX3 assembly in the *xz* plane. The ion impact positions *z* and *x* at the orthogonal TPX3 detector are determined by the flight angles θ and Φ of the ion beam leaving the pusher,^50^ which is given by
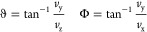
1where *v*_*x*_, *v*_*y*_, and *v*_*z*_ are the velocity components in *x*-, *y*-, and *z*-directions, respectively. We have observed that the TOF analyzer and LQ-trap parameters have a critical influence on *v*_*y*_ and *v*_*z*_ components of the ion beam, respectively, and hence on the θ, and this leads to a shift in the *z*-impact position at the orthogonal TPX3 detector. This will be discussed in detail below. As no voltage source accelerates the ions in the *x*-direction, *v*_*x*_ is much smaller than *v*_*y*_, which keeps Φ ≈ 90°; this means the *x*-impact position at the orthogonal detector is least sensitive to most of the ion optical parameters.

#### Orthogonal Ion Energy (*E*_*y*_)

**Figure 3:**
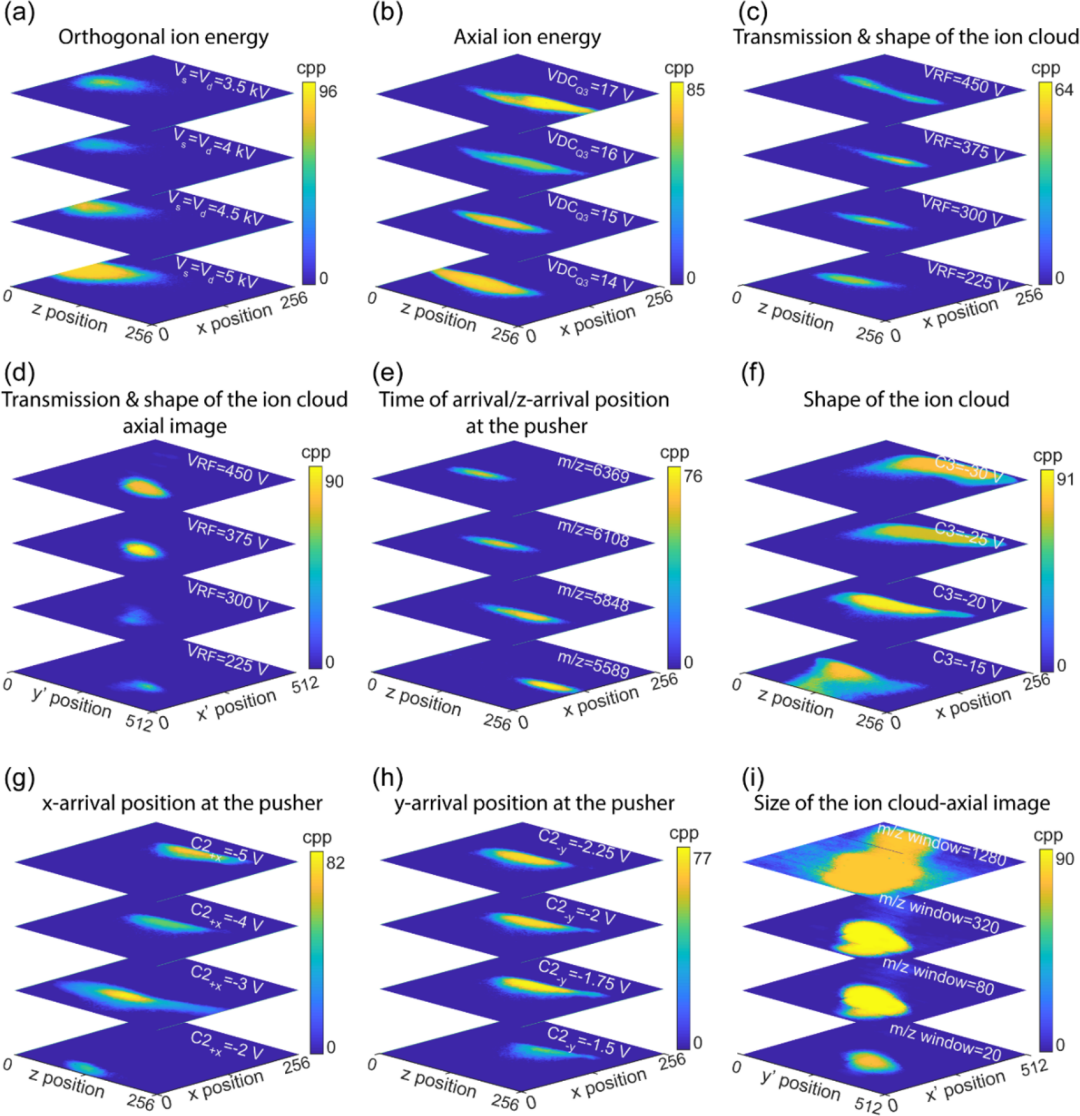
Influence of (a) s and d TOF fields (κ = 1), (b) DC voltage on the third segment of the LQ-trap (VDC_Q3_), (c) RF amplitude of the hexapole and LQ-trap (VRF), (e) *m*/*z* value, (f) voltage on the third electrode of the correction lens (C3), (g) voltage on the top segment of the second electrode of the correction lens (C2_+*x*_), (h) voltage on the left segment of the second electrode of the correction lens (C2_–*y*_) on the spatial distribution at the orthogonal TPX3 detector, and (d) VRF and (i) width of the *m*/*z* isolation window on the spatial distribution at the axial TPX quad detector. All data were collected by spraying the CsI mix and selecting singly charged ions with *m*/*z* of 5589 (except for (e)) using the quadrupole mass filter. All images represent the sum of a hundred measurement cycles. cpp = counts per pixel.

The impact of the TOF analyzer parameters on the *z*-impact coordinate of the orthogonal TPX3 image is shown in Figures 3a and S3a–d. The orthogonal velocity component (*v*_*y*_) of the ion beam as it enters the TOF analyzer is defined by the potential at the midpoint of *E*_1_ and *E*_2_ (Figure S3). This potential rises when (i) both *V*_s_ and *V*_d_ are increased while keeping κ as 1 (Figure 3a), (ii) *V*_s_ is increased at *V*_d_ = 5 kV (Figure S23a), and (iii) *V*_d_ is increased at *V*_s_ = 5 kV (Figure S23b), which leads to an increase of *v*_*y*_ in all three cases, raises θ, and results in a shift toward the −*z*-direction in the orthogonal TPX3 image. No significant *z*-shift in the ion profile was observed for a change in (i) *V*_s_ and *V*_d_ by retaining pusher voltage (*V*_s_ + *V*_d_) at 10 kV (Figure S23c) and (ii) bias electrode voltage while keeping *V*_s_ = 5 kV and *V*_d_ = 5 kV (Figure S23d) as all these changes do not significantly alter the potential profile at the midpoint of *E*_1_ and *E*_2_.

#### Axial Ion Energy (*E*_*z*_)

The DC offset voltage of the HCD cell (VDC_HCD cell_) defines the axial energy of the ion cloud as it leaves the HCD cell, but afterward, these ions are collisionally focused in the third segment of the LQ-trap (Q3) and ejected into the TOF region. Hence, the initial ion axial velocity (*v*_*z*_) when the beam enters the TOF region is determined by the DC component of the Q3 voltage (VDC_Q3_), not by the VDC_HCD cell_. An increase in *v*_*z*_ reduces θ, which causes a shift toward the +*z*-direction in the orthogonal TPX3 image. As expected, a rise in VDC_Q3_ results in a higher *v*_*z*_ velocity and shifts the ion trajectory more to the +*z*-direction (Figure 3b), whereas the *z*-impact position remains insensitive to the variations in the VDC_HCD cell_ (Figure S23f). The voltages of the adjacent electrodes of Q3-Q2 (second segment of the LQ-trap, Figure S23e) and L3 (Lens 3, Figure S23g,h) are also observed to influence *v*_*z*_. L3 is biased at a higher voltage during the ion storage in LQ-trap (*V*_L3 (Q-trapping)_) and switches to a lower value when the ions from the LQ-trap are ejected into the TOF region (*V*_L3 (Ejection to pusher)_). Changes in *V*_L3 (Q-trapping)_ and *V*_L3 (Ejection to pusher)_ have totally opposite effects on the *z*-impact position of the ion distribution at the orthogonal TPX3 image. Supporting Information provides a detailed description on the influence of Q2 and L3 on the axial ion energy and consequently on the spatial profile at the orthogonal imaging detector (Supporting Information Section “Influence of Ion Optics on Orthogonal Spatial Profile” and Figure S23e,g,h).

#### Transmission and Collisional Focusing of the Ion Cloud

The effect of RF amplitude of the hexapole and LQ-trap (VRF) on the ion transmission has already been explored by evaluating the Orbitrap spectrum acquired in the external Orbitrap mode (Supporting Information, Section “Characterization of the Orbitrap/TOF Instrument without Imaging Detectors and UV Laser” and Figure S19). Here, we investigated the spatial profile of the quadrupole-isolated ions with *m*/*z* values of 5589 (Figure 3c,d), 3510.5 (Figure S23i,j), and 7407.7 (Figure S23k,l) using the orthogonal TPX3 and axial TPX quad detectors at different VRF values. The axial TPX quad images indicate that higher VRF is an absolute requirement for the effective transmission of high *m*/*z* ions. The utilization of VRF = 300 V was sufficient for the efficient transmission of *m*/*z* = 3510.5 ions. Meanwhile, a VRF of 375 V was necessary for *m*/*z* = 5589 and 7407.7 ions. The orthogonal TPX3 images provide a clearer picture of the VRF dependency on the *m*/*z*. For instance, the shape of the ion cloud remains the same or the ion trajectory stabilizes when VRF > 300 V for *m*/*z* = 3510.5 ions. However, the ion trajectories do not stabilize even when the VRF approaches 450 V for ions with *m*/*z* = 5589 and 7407.7.

#### *m*/*z* Dependency

A previous study by our group conducted on the TPX quad-equipped LCT (ESI-orthogonal reflectron TOF) demonstrated that the centroid of the spatial distribution of the ion cloud at the TPX detector is insensitive to *m*/*z* values.^50^ Despite the fact that both the LCT and Orbitrap/TOF instruments utilize ESI sources that produce a continuous ion beam, the centroid of the *m*/*z* resolved images acquired on the Orbitrap/TOF instrument shifts in the *z*-direction (Figure 3e). In LCT, a continuous ion beam produced by the ESI source is pulsed by the pusher toward the detector, causing ions to strike at the same detector area regardless of the *m*/*z* values, whereas in the Orbitrap/TOF instrument, the ESI-generated ions are later collisionally focused in the LQ-trap. This well-focused discrete ion packet pulsed from the LQ-trap is *m*/*z* separated in the time domain (*z*-position) while traveling the substantial distance from the LQ-trap to the pusher. This causes the fast-moving low *m*/*z* ions to appear more to the right of the pusher (more toward the +*z*-direction) compared to the slow-moving high *m*/*z* ions, prior to the pulsing of the ion cloud to the orthogonal detector. Figure S24a further supports this explanation. The dependency of the spatial distribution of the ion cloud at the orthogonal detector on the time difference between the ion ejection from the LQ-trap and pusher pulse (*T*_Pusher pulse_ – *T*_Ejection from LQ-trap_) is depicted in Figure S24a. At a lower *T*_Pusher pulse_ – *T*_Ejection from LQ-trap_, the ions appear more to the left of the pusher prior to the pulsing and are imaged more to the −z-direction of the detector. When the *T*_Pusher pulse_ – *T*_Ejection from LQ-trap_ is higher, the ions have the time to travel further to the right of the pusher and are more likely to strike the detector toward the +*z*-direction.

#### Focusing and Deflection of the Ion Beam

Figures 3f and S24b,c demonstrate the dependency of the spatial profile of the ion cloud at the orthogonal TPX3 detector on the applied voltages on the third, first, and second electrodes of the correction lens (C1–3). The correction lens positioned between the LQ-trap and pusher focuses and defocuses the ion beam based on the applied voltages on the three electrodes, which leads to a change in the shape as well as a shift in the centroid (*z̅*, *x̅*) of the ion cloud that is projected onto the orthogonal detector. Note that the variations in the voltage of the electrode near the exit of the correction lens, lens 4 (L4), have an impact on the shape of the ion cloud as well (Figure S24d). The middle electrode of the correction lens, C2, has the least effect on the ion beam spatial profile of the three electrodes. C2 is a segmented four-electrode steering lens. The voltage of each segment can be adjusted separately to function as a *xy* deflector. A change in the *x*-deflection voltage (*V*_+*x*_) of C2 alters the *x*-position of the ions at the pusher region prior to pulsing and leads to a considerable shift in the *x*-impact coordinate of the ion cloud at the TPX3 detector image (Figure 3g). Similarly, a variation in the *y*-deflection voltage (*V*_–*y*_) affects the *y*-position of the ions at the pusher region prior to pulsing as well. However, the modification of the ion trajectories in the *y*-direction is not translated to the ion image as the orthogonal detector is placed in the *xz* plane (Figure 3h).

#### Size of the Ion Cloud

Figure 3i shows the effect of the *m*/*z* isolation window on the axial TPX quad detector image. At high *m*/*z* isolation window values, a large ensemble of ions is transported from the quadrupole of the Orbitrap MS to LQ-trap, which results in increased Columbic repulsion within the ion packet, and it expands in all directions. An *x*–*y* slit (Element 17, Figure 2) of 2 × 12 mm cuts the collisionally focused ion cloud ejected from the LQ-trap as a rectangular beam. The ion cloud is then enlarged further as it travels through the field-free zone between the slit and exit of the pusher. A tilted, distorted rectangular-shaped ion cloud is observed at the axial imaging detector as the rectangular TPX quad chip is positioned at an angle in the *xy* plane. The dimensions of the ion cloud are measured as ∼2 × 3, 2.2 × 5.4, 2.2 × 6.3, and 2.3 × 11 mm (*x*–*y*) at different *m*/*z* isolation windows of values 20, 80, 320, and 1280, respectively.

### Characterization of the Orbitrap/TOF Instrument with External UVPD Imaging

In this section, the implementation of UVPD using the 193 nm excimer laser on the Orbitrap/TOF instrument equipped with the orthogonal imaging detector is discussed. High-resolution measurements in external UVPD Orbitrap mode were initially performed followed by the *m*/*z*-resolved spatial distribution determination in external UVPD orthogonal TOF imaging mode of the UV-generated fragments from the MMA. The UVPD experiments were conducted in the System configuration 2 (Figures 1a and 2) as the axial UVPD implementation in Configuration 2 enables maximum overlap of the photons with the ion cloud. Concanavalin A (102 kDa), a noncovalently bounded homotetramer, was injected into the MS under native-like conditions. The tetrameric concanavalin A [M + 21H]^21+^ ions were selected with an *m*/*z* window of 10 Da using the quadrupole mass filter. The laser beam interacts with the selected precursor ions at one of the locations in between the LQ-trap and pusher, and the UV fragments are then sent either to the Orbitrap analyzer or to the orthogonal TPX3 detector.

#### High-Resolution UVPD

**Figure 4:**
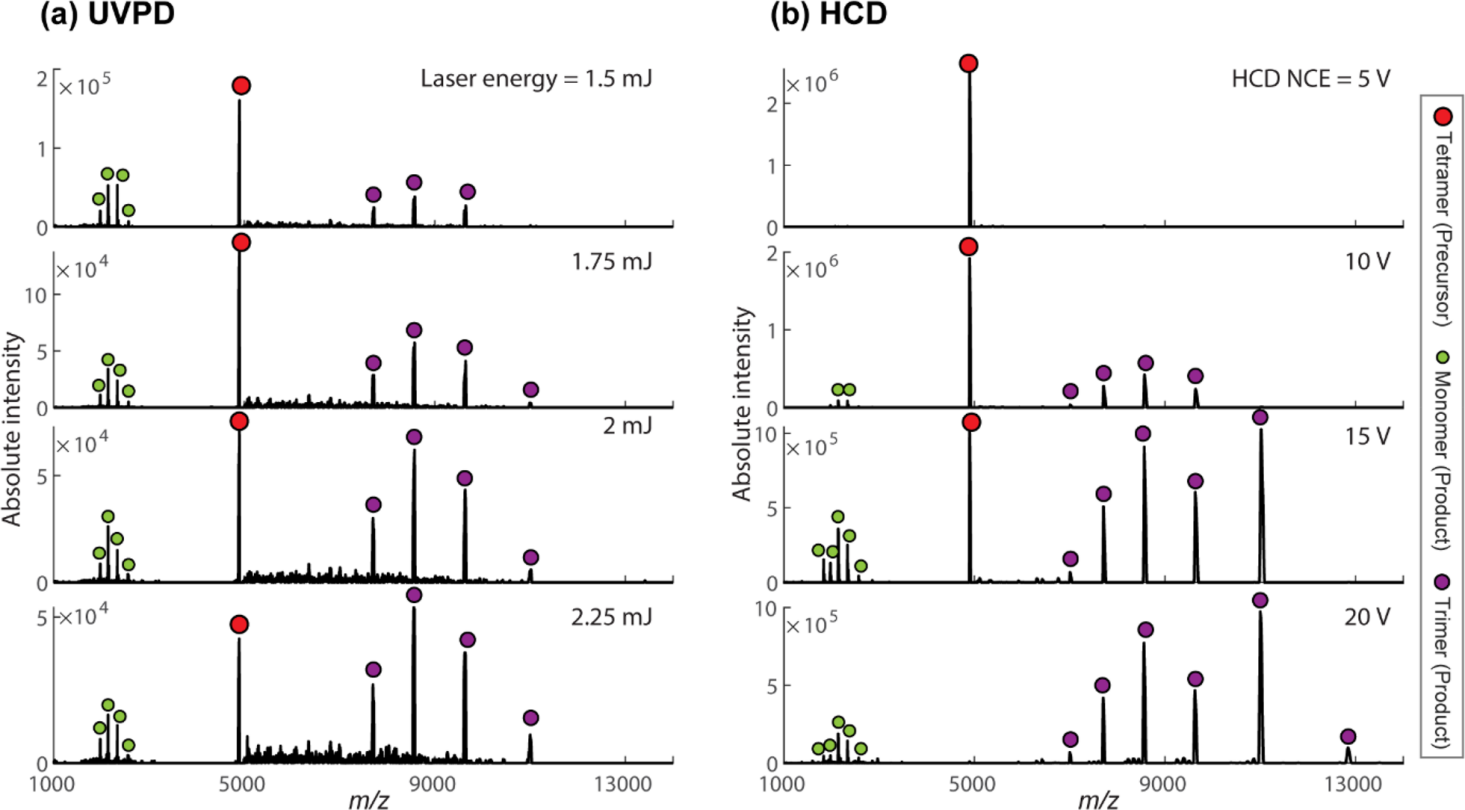
(a) UVPD spectra of tetrameric concanavalin A [M + 21H]^21+^ ions acquired at different laser pulse energies in external UVPD Orbitrap mode. A single UV laser pulse interacts with the precursor ions in the LQ-trap for 100 ms (ion storage time) per measurement cycle. (b) HCD spectra of the tetrameric concanavalin A [M + 21H]^21+^ ions measured at various collision energies (normalized collision energy, NCE). Both the UVPD and HCD spectra were recorded under the same Orbitrap MS settings. Table S3 and Figures S14 and S25 show the important data acquisition parameters and event sequence used in external UVPD Orbitrap mode.

Figure 4a shows the UVPD spectra of tetrameric concanavalin A [M + 21H]^21+^ ions acquired at different laser pulse energies in external UVPD Orbitrap mode. In this mode, a single UV laser pulse interacts with the precursor [M + 21H]^21+^ ions in the LQ-trap for 100 ms (ion storage time) per measurement cycle. The generated UV fragments were sent back to the Orbitrap analyzer for high-resolution molecular analysis. In Figure 4b, the HCD spectra of the tetrameric concanavalin A [M + 21H]^21+^ ions measured at various collision energies (normalized collision energy, NCE) are displayed for better comparison. Both the UVPD and HCD spectra were recorded under the same Orbitrap MS settings. Table S3 and Figures S14 and S25 show the relevant data acquisition parameters and event sequence used in external UVPD Orbitrap mode. The UVPD data indicates that the tetramer predominantly dissociates into monomers and trimers, in line with the results from the HCD and previous UVPD^40^ measurements. As the laser pulse energy increases, the signal intensity of the precursor peak reduces as expected. However, fragment peak intensities do not significantly increase. This can be attributed to the following reasons: (i) ion beam divergence due to the increased space-charge effect; (ii) insufficient cooling of the UV-generated fragments in the LQ-trap. The ratio of monomers to trimers is low in our study when compared to the UVPD spectrum of the concanavalin A [M + 21H]^21+^ ions obtained by the interaction of 3 mJ, 193 nm photons at the HCD cell in previous investigations.^40^ This can be attributed to the discrepancy between the UV dissociation conditions and data acquisition parameters used in the two experimental setups. However, we believe that, in our case, the high RF voltage (VRF = 600 V) and the low RF frequency (515 Hz) of the hexapole and LQ-trap may contribute to the preferential transmission of the high *m*/*z* trimer product ions. Note that a higher VRF (>450 V) and a lower LQ-trap pressure (<1 × 10^–4^ mbar) were essential requirements for the measurement of UVPD fragments in external Orbitrap UVPD mode.

#### UVPD Fragment Orthogonal TOF MS Ion Imaging

**Figure 5:**
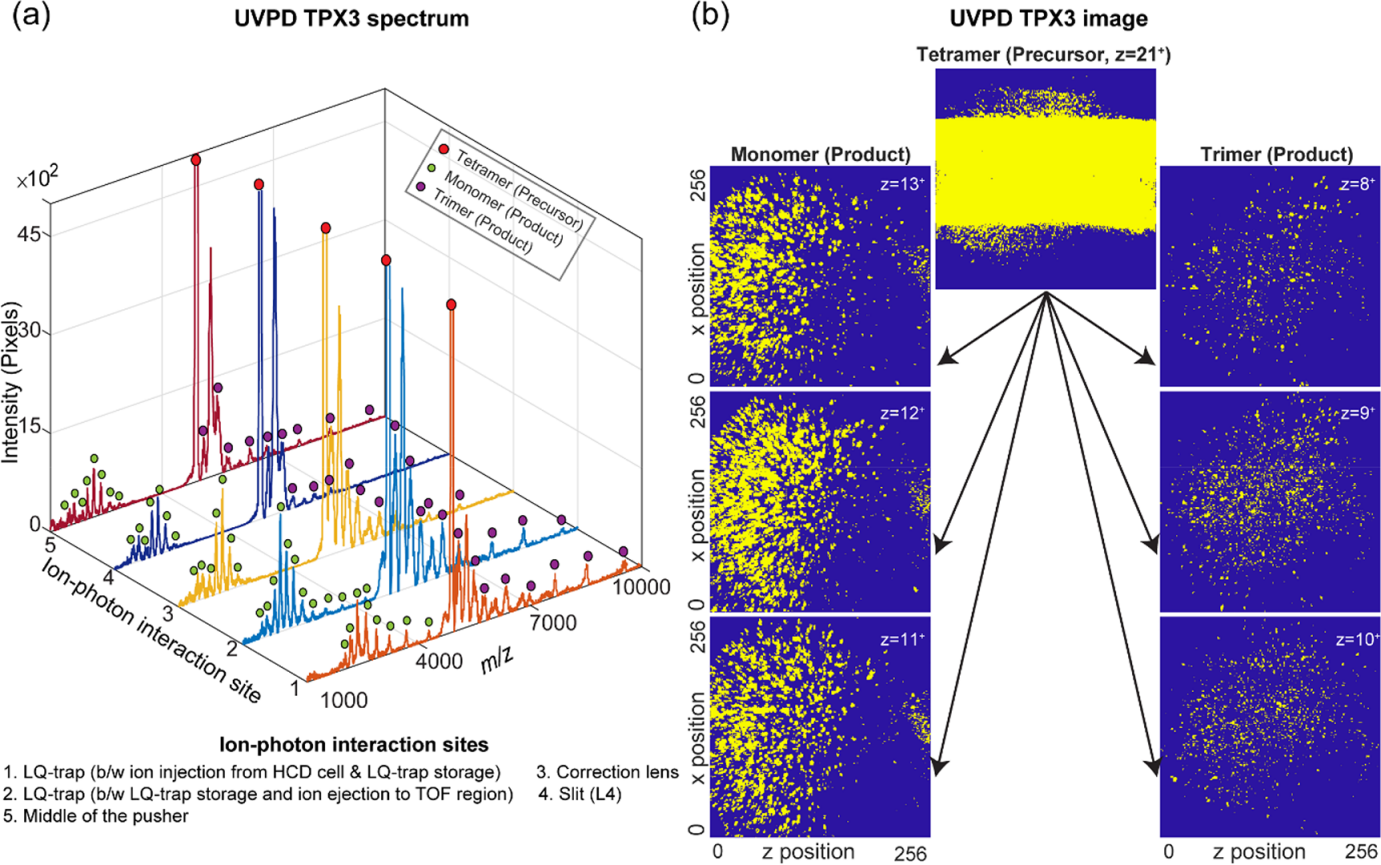
(a) UVPD spectra of tetrameric concanavalin A [M + 21H]^21+^ ions acquired at the orthogonal TPX3 detector by the interaction of 193 nm photons with the ion cloud at different locations of the instrument. (b) Spatial distribution of the product ions and unfragmented precursor ions at the TPX3 detector generated by the photon–ion interaction close to the middle of the TOF region (image corresponds to maroon trace in (a)). 21^+^ charged precursor ion cloud dissociates predominantly into fragment ions that are 13^+^ charged monomers and 8^+^ charged trimers, 12+ charged monomers and 9^+^ charged trimers, or 11^+^ charged monomers and 10^+^ charged trimers. A single laser pulse with an energy of 0.5 mJ (measured at the exit of the attenuator) per TOF cycle was used. A hundred TOF measurement frames were added to produce the total ion spectrum. Table S4 and Figures S16 and S26 show the important data acquisition parameters and event sequence used in external UVPD orthogonal TOF mode.

The UVPD fragments can also be studied with the orthogonal TOF MS. Figure 5a shows the UVPD spectrum of the concanavalin A [M + 21H]^21+^ precursor ions measured at the orthogonal TPX3 detector by the interaction of 193 nm photons with the ion cloud at different locations of the instrument (Elements 14–18, Figure 2). A single laser pulse with an energy of 0.5 mJ (measured at the exit of the attenuator) per TOF cycle was used. A hundred TOF measurements were added to produce the total ion spectrum. Table S4 and Figures S16 and S26 show the relevant data acquisition parameters and event sequence used in external UVPD orthogonal TOF mode. In all five cases, tetramer is fragmenting to monomers and trimers, which is consistent with the results of high-resolution UVPD. The orange trace (Figure 5a) corresponds to the photon–ion interaction right after the precursor ion injection from the HCD cell to LQ-trap. The light blue trace displays the result of the interaction of the laser with the precursor ions, stored in the LQ-trap for 100 ms, just before the ion ejection into the TOF analyzer region. The yellow, dark blue, and maroon traces represent the UVPD spectra generated by the interaction of the laser beam with the moving precursor ion cloud at different locations. These are, respectively, the correction lens (Element 16, Figure 2), the slit (Element 17, Figure 2), and the middle of the TOF analyzer (Element 18, Figure 2). The interaction locations were predicted by comparing the experimentally measured time between the ion injection from the HCD cell to LQ-trap and the pusher pulsing with the Simion ion optics model. The orange, light blue, and yellow UVPD spectra were plotted by combining the TPX3 spectrum collected for six different *T*_Pusher pulse_ – *T*_Ejection from LQ-trap_ values (time taken by the ion cloud to travel from the LQ-trap to the middle of the TOF analyzer) of 140, 160, 180, 200, 220, and 240 μs. The *T*_Pusher pulse_ – *T*_Ejection from LQ-trap_ had to be tuned to 200 μs to ensure that the precursor [M + 21H]^21+^ (*m*/*z* = 4901) ions strike at the center of the imaging detector assembly. However, when UVPD takes place at a location prior to the middle of the pusher, the ion beam will be axially dispersed (in the *z*-direction) based on the *m*/*z* values of the product ions, when they arrive at the pusher. A lower *T*_Pusher pulse_ – *T*_Ejection from LQ-trap_ (∼140 to 180 μs) for the fast-moving monomer product ions that span over a lower *m*/*z* range from 1700 to 4300 Da and a higher *T*_Pusher pulse_ – *T*_Ejection from LQ-trap_ (∼200 to 240 μs) for the slow-moving trimer product ions that span over a higher *m*/*z* range from 5100 to 9700 Da had to be used in order to ensure that the ions in the entire *m*/*z* range strike at the MCP detector area (40 mm diameter). The effect of the axial velocity spread of the product ions is minimized when the ion–photon dissociation location is close to the midpoint (*z*-component) of the pusher, where the pusher pulses the ions toward the TPX3 detector. A single measurement with *T*_Pusher pulse_ – *T*_Ejection from LQ-trap_ of 200 μs was adequate to encompass the whole *m*/*z* range of the UVPD spectrum, when the ion–photon interaction occurs at the midpoint of the pusher region (maroon curve, Figure 5a). The dark blue curve is generated by the interaction of the laser beam with precursor ions at the slit using a single *T*_Pusher pulse_ – *T*_Ejection from LQ-trap_ of 200 μs. Several product ions (mainly high *m*/*z*’s) were not detected in this case due to the axial separation as they move through a potential gradient of 40 V from the slit to the middle of the pusher (65 mm). To minimize the TOF effect that results in the axial dispersion of the product ions, the UVPD fragment imaging experiments were performed by the interaction of the UV laser beam with the precursor ion cloud at the midpoint of the pusher.

The spatial distribution of the product ions and unfragmented precursor ions at the orthogonal TPX3 detector generated by the photon–ion interaction close to the middle of the pusher is shown in Figure 5b (image corresponds to maroon trace in Figure 5a). A well-focused 21^+^ charged precursor ion cloud dissociates predominantly into fragment ions that are 13^+^ charged monomers and 8^+^ charged trimers, 12^+^ charged monomers and 9^+^ charged trimers, or 11^+^ charged monomers and 10^+^ charged trimers, which spread all over the detector area in both *x*- and *z*-directions. The monomer and trimer product ions are unambiguously separated in both time and space. While outside the scope of this study, a better understanding of the kinetics of the dissociation process can be gained by analyzing the relative distance and angular distribution of the product ions with respect to the impact position of the precursor MMA ions, which may reveal critical details about the higher order structural characteristics of the MMA such as bond strength and 3D conformation.

## Conclusions and Outlook

With the development of a unique Orbitrap/TOF system with integrated UVPD and TPX3CAM, we have brought together aspects from high-resolution Orbitrap and TOF MS, top-down proteomics, and photofragment ion imaging for the first time. This paves the way for an entirely new approach for resolving the higher-order molecular structure of MMAs in their pseudonative state in the gas phase. The custom-developed instrument, which is operational in four different modes, enables the high mass resolution measurement and mass-resolved imaging of the UVPD-generated fragments from the native MMA ions using the Orbitrap mass analyzer and TOF analyzer-TPX3 imaging assembly, respectively.

UVPD on high-resolution Orbitrap and TOF MS instruments has already been employed by several groups for the high-level structural and functional characterization of MMAs.^36,40,78−82^ However, the TOF imaging approach implemented in this study with the TPX3 detection assembly allows the visualization of the 3D UV dissociation event of the MMA’s as the TOF analyzer is designed to maintain the relative positions of the fragment subunits until reaching the detector. A better understanding of the dissociation dynamics can be gained through the analysis of the relative distance and angular distribution of the product ions with respect to the impact position of the precursor MMA ions from the *m*/*z*-resolved TPX3 images. We hypothesize that this approach will provide crucial information regarding the higher-order structural characteristics of the MMA, including bond strength, conformation, etc. as well as the behavior of the MMAs in the gas phase. In addition, the evaluation of the *m*/*z*-resolved TPX3 images after the integration of other fragmentation methods such as ECD, ETD, SID, and IRMPD to the Orbitrap/TOF system is anticipated to yield significant information on different fragmentation mechanisms.

Previous studies conducted with the MCP-TPX quad equipped LCT (nESI-orthogonal reflectron TOF MS) system demonstrated the capability of the TPX detector family to detect noncovalent protein complexes and to image single ion events.^50,52^ The utilization of the single ion sensitivity of the TPX3 detection assembly and the mass separation of the UVPD subunits generated from the MMA while maintaining the relative positions in the TOF analyzer in this instrument may provide the TPX3 images that can be used to obtain the 3D geometry of the MMA, when the UVPD occurs at the level of the single precursor MMA ion.

## Supporting Information Available

Ion optics design of TOF analyzer; characterization of the Orbitrap/TOF instrument without imaging detectors and UV laser; influence of ion optics on orthogonal spatial profile of the ion cloud; data acquisition parameters and event sequences (Tables S1–S4, Figures S14–S18, S22, and S25–S26); schematic of the configuration 1b of the Orbitrap/TOF system (Figure S1); detailed ion and laser optics and schematic of configuration 1b of the Orbitrap/TOF system (Figure S2); ion optics schematic of the TOF analyzer (Figure S3); projections of the simulated isotropic distribution of the concanavalin A and ubiquitin fragments (Figure S4); effect of the TOF voltage parameters on time focus (Figures S5–S6); simulated ion trajectories of the ubiquitin and concanavalin A fragments at different TOF voltage parameters and fragmentation locations (Figures S6–S10); simulated detector images and TOF spectra of the concanavalin A and ubiquitin fragments collected at the orthogonal detector at different dissociation energies and locations (Figures S11–S12); CsI spectra acquired by operating the Orbitrap/TOF instrument at different modes in configuration 1a (Figure S13); effect of ion optics parameters on the *m*/*z* profile (external Orbitrap mode, Figures S19–S21); influence of ion optics on the orthogonal spatial profile of the ion cloud (Figures S23–S24) (PDF)
