# Supplementary material for: An Orbitrap/Time-of-Flight Mass Spectrometer for Photofragment Ion Imaging and High-Resolution Mass Analysis of Native Macromolecular Assemblies
Source: J Am Soc Mass Spectrom. 2023 Jun 15;34(7):1359–71. doi: 10.1021/jasms.3c00053 (PMC10326918; doi:10.1021/jasms.3c00053)
Supplement: Supplementary file 1 — js3c00053_si_001.pdf [file js3c00053_si_001.pdf]

Supplementary Material for

An Orbitrap/Time-of-Flight Mass Spectrometer for Photofragment Ion Imaging and High Resolution Mass Analysis of Native Macromolecular Assemblies

Anjusha Mathew[1], Frans Giskes[1], Alexandros Lekkas[2], Jean-François Greisch[3,4], Gert B. Eijkel[1], Ian G. M. Anthony[1], Kyle Fort[5], Albert J. R. Heck[3,4], Dimitris Papanastasiou[2], Alexander A. Makarov[3,5], Shane R. Ellis[1,6*], and Ron M. A. Heeren[1*]

[1]Maastricht MultiModal Molecular Imaging (M4i) Institute, Division of Imaging Mass Spectrometry (IMS), Maastricht University, 6229 ER Maastricht, The Netherlands

[2]Fasmatech Science and Technology, Demokritos NCSR, 15310 Agia Paraskevi, Athens, Greece

[3]Biomolecular Mass Spectrometry and Proteomics, Bijvoet Centre for Biomolecular Research and Utrecht Institute for Pharmaceutical Sciences, Utrecht University, Padualaan 8, 3584 CH Utrecht, The Netherlands

[4]Netherlands Proteomics Center, Padualaan 8, 3584 CH Utrecht, The Netherlands

[5]Thermo Fisher Scientific (Bremen) GmbH, 28199 Bremen, Germany

[6]Molecular Horizons and School of Chemistry and Molecular Bioscience, University of Wollongong, NSW 2522, Australia

*To whom correspondence should be addressed:

r.heeren@maastrichtuniversity.nl

sellis@uow.edu.au

Ion Optics Design of TOF analyzer

This section describes the ion optical design and simulations of the TOF analyzer. The TOF analyzer and the associated ion optics of the custom-designed system was modelled using SIMION and SIMAX. An isotropic spherical expansion of some of the expected covalently/non-covalently dissociated fragments from ubiquitin (8.6 kDa) and dimeric concanavalin A (51 kDa) was generated for the simulations, where the velocity vectors of the fragments were determined by assuming a kinetic energy (E) release of 2 to 5 eV/fragment ($E_{Fragmentation}$) following the dissociation (Supporting Information Figure S4). The ion beam was considered to be initially accelerated in the axial (z) direction by 30 V (V_{axial}). The precursor ubiquitin ion of *m/z*=1428, z=6 and axial kinetic energy (E_{Axial})=180 eV dissociates either into two fragment ions of mass=4.28 kDa (*m/z*=1428, z=3) or fragment ions of masses 1.25 kDa (*m/z*=1247, z=1) and 7.31 kDa (*m/z*=1463, z=5). While the precursor concanavalin A homodimer of *m/z*=3422, z=15 and E_{Axial}=450 eV dissociates into two monomer fragment ions of *m/z*=3209 (z=8) and *m/z*=3667 (z=7).

Figure S5 demonstrates the effect of V_s, V_d and bias electrode voltage ($V_{bias\ electrode}$) on the time focus at a nominal acceleration potential of 15 kV (pusher voltage). The time focus value displayed in the y-axis was calculated by measuring the distance from the pusher to the TOF region with the minimum spatial spread of the ion beam in y (orthogonal)-direction. κ is the ratio of V_s to V_d. A concanavalin A $[M+8H]^{8+}$ monomer fragment ion cloud formed at the midpoint of the pusher was used for the determination of time focus. The time focus has observed to be least sensitive to the $V_{bias\ electrode}$ at higher κ values. At higher κ values, for example, when κ=0.5 (V_s=5 kV and V_d=10 kV), the time focus drifts only by a maximum distance of 130 mm for a change in $V_{bias\ electrode}$ from -15 to +15 kV (Figure S6). Figures S7 and S8 show the ion trajectories (in xy plane) of the concanavalin A $[M+8H]^{8+}$ and $[M+7H]^{7+}$ monomer fragment ion clouds formed at the midpoint of the pusher, correspond to different $V_{bias\ electrode}$ at two relatively high κ values, κ = 0.364 (V_s=4000 V, V_d=11000 V) and 0.5, respectively. Note that the relative positions of the fragment ions following the dissociation process are maintained at both κ values. However, when the fragmentation occurs in the middle of the second segment of the correction lens (C2) and κ = 0.364, the ion trajectories cross on their way to the detector and results in the distortion of the relative positions of the ions at the detector image (Figure S9). Whereas, the relative positions of the ions are preserved regardless of the fragmentation location when κ = 0.5 (Figure S10). In addition, the angular divergence of the fragments is least sensitive to the variations in $V_{bias\ electrode}$ at κ=0.5 compared to 0.364. The simulation results suggest that the optimum conditions for the operation of the instrument in the external UVPD orthogonal TOF imaging mode are the following: κ ≥ 0.5, distance from the pusher to the detector=655 mm and the fragmentation location at the

center of the pusher. In order to enhance the time resolution and maximize the number of ions striking the detector, V_s, V_d and $V_{bias\ voltage}$ should be fine tuned.

Figures S11 and S12 show the simulated total ion images and TOF spectra collected at the orthogonal detector at different dissociation energies and locations by sending concanvalin A and ubiquitin ions, respectively. There is a shift in the z-impact position of the ion cloud at the detector image (Figures S11 and S12a, c, e and g) based on the *m/z* that is caused by the difference in the flight angle (θ). θ is defined as the ratio of orthogonal (y) and axial (z) velocity components. The product ions have the same v_z as the precursor ions regardless of the *m/z* values (neglecting the energy redistribution during the fragmentation process). In contrast, as all ions are accelerated with the same kinetic energy per charge in the TOF analyzer resulting in higher v_y for low *m/z*s and low v_y for high *m/z*s. This causes $\theta_{low\ m/z} > \theta_{high\ m/z}$. The reduced θ angle causes the high *m/z* ion beam to strike more towards the left of the detector. The detector required to be off-centered in the z-direction by ~20 mm when operated with a nominal acceleration potential of 15 kV because of the shift in the z-impact position due to the flight angle and to prevent the ions from travelling through the TOF region with non-straight equipotential lines. This has been considered during the mechanical design, and the detector assembly flange is constructed in such a way that it can be shifted by a maximum of 40 mm in z-direction.

The effect of fragmentation location, $E_{Fragmentation}$ and *m/z* on the detector images as well as TOF spectra are evident in Figures S11 and S12. Even though the TOF spectra have improved time resolution when the fragmentation occurs in C2 and C3 (Figures S11 and S12b and d), the shape of the ion cloud (FigureS S11 and S12a and c) is distorted due to the axial fragment ion acceleration following the dissociation process. Moreover, the ion transmission is significantly affected in both cases because of the slit (Element 17, Figure 2) that is positioned between the correction lens and TOF analyzer, which cuts the ion beam in y-direction. This also suggests that the higher time resolution in Figures S11 and S12b and d compared to f and h is due to the absence of the fragments with high angular spread in the y-direction. In Figures S11 and S12f and d, all of the fragments including the one with high angular spread, reach the detector and result in poorer quality TOF spectra as the fragmentation occurs at the pusher. However, this time resolution is still adequate to distinguish the complementary fragment pairs generated from single MMA ions in external UVPD orthogonal TOF imaging mode. Also note that detector image (xz plane) in Figures S11 and S12e and g resembles more to the projection of the initial distribution of the ion cloud (Figures S4e,f), demonstrating once again that relative positions of the ions are better retained when fragmentation occurs in the pusher region.

Characterization of the Orbitrap/TOF Instrument without Imaging Detectors and UV Laser

To experimentally characterize the performance of the custom designed TOF analyzer and the associated transmission ion optics, two discrete-dynode EMs were connected axially and orthogonally to the TOF analyzer instead of the imaging detectors. As this instrument is intended for the investigation of high mass ions, cesium iodide (CsI) mix was sprayed due to its high and wide *m/z* range (392 to 11,304 Da), and the signal was analyzed in all modes of operation under identical ion optical conditions (Figure S13). The important data acquisition parameters and event sequences used in different modes of operation are shown in Table S1 and Figures S14-18. Spectra recorded in the external Orbitrap mode and standard Orbitrap mode are very similar, as expected. In external axial non-imaging mode, the CsI signal is measured without distinguishable spectral information due to the lack of mass analyzer between the LQ-trap and axial discrete-dynode EM detector. The CsI signal measured at the axial discrete-dynode EM detector has therefore been determined as a function of arrival time. The spectrum measured in external orthogonal TOF non-imaging mode, resembles the standard Orbitrap spectrum in terms of the *m/z* peaks identified. Although the mass resolution in TOF mode is only 327 at *m/z* = 3510, that still adequately resolves the adjacent peaks in the high *m/z* CsI mix spectrum. In addition, there is a slight difference in the relative intensities of each of the *m/z* peaks in Orbitrap and TOF spectrum. For example, the base peak in Orbitrap spectrum is *m/z*= 2471, while the base peak in TOF spectrum has a higher *m/z* value of 3510. The difference in the intensity profiles can be attributed to the *m/z* dependency of the arrival time of the ions at the pusher region. The fast moving-low *m/z* ions appear more to the right of the pusher (Figure 2) compared to the slow moving high *m/z* ions prior to the pulsing of the ion cloud to the orthogonal detector. At a lower $T_{Pusher\ pulse}$-$T_{Ejection\ from\ LQ\text{-}trap}$ (time difference between the ion ejection from LQ-trap and pusher pulsing, Figure S18), for example at 50 µs, the TOF spectrum is dominated by low *m/z* ion signal, as most of the high *m/z* ions appear to be lost on the left side of the detector. Whereas, when the $T_{Pusher\ pulse}$-$T_{Ejection\ from\ LQ\text{-}trap}$ is high, for example at 350 µs, mostly high *m/z* ions appear in the spectrum, as they get sufficient time to reach the pusher zone before being pulsed to the detector area. The majority of the low *m/z* ions is not measured in this case as they are lost on the right side of the detector. For the acquisition of the TOF spectrum in Figure S13, $T_{Pusher\ pulse}$-$T_{Ejection\ from\ LQ\text{-}trap}$=270 µs was used that prefer mostly the high *m/z* ions to be pulsed to the detector area.

We have observed that only a few ion optical parameters associated with the custom designed part of the instrument have critical influence on the resulting spectrum: RF amplitude of the hexapole and LQ-trap, trapping gas pulse width and time delay between

Ar gas injection to the LQ-trap and the ion ejection from the HCD cell to LQ-trap. The effect of the aforementioned parameters on the *m/z* profile is briefly discussed below.

RF amplitude of the hexapole and LQ-trap: Figure S19 shows the denatured ubiquitin spectrum measured in external Orbitrap mode at different RF amplitude of the hexapole and LQ-trap (VRF). An increase in the RF amplitude shifts the *m/z* transmission window to higher *m/z* range.[1-2] When VRF is low, for example, at VRF=50 V, the mass spectrum is dominated by low *m/z* ions with a z=13+ to 6+. Whereas, at a higher VRF=350 V, mostly lowly charged (9+ to 4+) high *m/z* ions appear at the mass spectrum.

Trapping gas pulse width: Figure S20 demonstrates the effect of the amount of trapping gas (Ar) injected to the LQ-trap (Ar gas pulse width) on the mass spectrum. Denatured ubiquitin was measured in the external Orbitrap mode. At a pulse width of 60 µs, the amount of Ar gas required to collisionally focus the ion cloud in the LQ-trap was insufficient that resulted in reduced signal intensity. A change in the gas pulse width from 60 to 70 µs, increased the ion intensity by three fold. The ion intensity continues to increase until the pulse width reaches 75 µs. Even if the pulse width is increased to a value as high as 100 µs, there is no significant change in ion intensity after 75 µs. A higher value of Ar gas pulse width was required for the efficient trapping of high molecular weight (MW) species. For instance, a minimum pulse width of 80 µs was required for the sufficient trapping and focusing of tetrameric Concanvalin A with a MW of 102 kDa in the LQ-trap.

$T_{\text{Ar gas injection to LQ-trap - Ion injection to LQ-trap}}$: The impact of the $T_{\text{Ar gas injection to LQ-trap - Ion injection to LQ-trap}}$ (time delay between the Ar gas injection into the LQ-trap and ion ejection from HCD cell to the LQ-trap) on the Orbitrap spectrum of denatured ubiquitin is depicted in Figure S21. Table S2, Figures S15 and S22 shows the important parameters and event sequence used for the acquisition of Figures S19-21. All of the left sided spectra (Figure S19a, c and e) were recorded in the external Orbitrap mode. All of the right sided Orbitrap spectra (Figure S21b, d and f) were collected by sending the ions from the HCD cell to the LQ-trap and storing them there instead of sending back to the Orbitrap MS by applying a high voltage of 50 V to lens 1. Ideally, the left sided Orbitrap spectrum is expected to have a maximum intensity of 10^6 (maximum intensity measured in standard Orbitrap mode) and the right one should not contain any signal at all. At $T_{\text{Ar gas injection to LQ-trap - Ion injection to LQ-trap}}=2$ ms, the base peak of the left spectrum has the same intensity as the base peak of the standard Orbitrap mode spectrum, and very few ions were measured in the right spectrum, indicating that most of the ions are efficiently transmitted from the HCD cell to LQ-trap. When $T_{\text{Ar gas injection to LQ-trap-Ion injection to LQ-trap}} = 0$ ms, the ion cloud is poorly trapped in the LQ-trap as there is no time between the Ar gas and ion injection into the LQ-trap (Figure S21a). Whereas, at a higher $T_{\text{Ar gas injection to LQ-trap-Ion injection to LQ-trap}}=27$ ms, the Ar gas expand to more ion optics components placed in between the LQ-trap and HCD cell that prevents the ion cloud leaving from the HCD cell and reaching the LQ-trap. This leads to the

detection of the high intensity mass spectrum in both cases as the ions are not even leaving the Orbitrap MS (Figure S21e and f). $T_{\text{Ar gas injection to LQ-trap-Ion injection to LQ-trap}}$ between 1 and 20 ms is recommended for the optimum operation of the instrument.

Influence of Ion Optics on Orthogonal Spatial Profile

Axial ion energy (E_z): Despite the fact that the defined potential well is at Q3, ions may be oscillating between the second segment of the LQ-trap (Q2) and Q3 during the collisional cooling of ion cloud in the LQ-trap. This means that the DC component of Q2 voltage (VDC_{Q2}) also likely to have an impact on v_z. A shift towards +z direction has been observed with an increase of VDC_{Q2} (Figure S23e). The lens 3 (L3), the electrode placed next to the Q3, is also observed to have an effect on v_z. L3 is at a higher voltage during the ion storage in LQ-trap ($V_{L3\ (Q\text{-trapping})}$) and switches to a lower value when the ions from the LQ-trap are ejected to the TOF region ($V_{L3\ (Ejection\ to\ pusher)}$) (Figure S16). A change in $V_{L3\ (Q\text{-trapping})}$ and $V_{L3\ (Ejection\ to\ pusher)}$ have opposite effects on the z-impact position of the ion distribution at the orthogonal TPX3 image. A higher $V_{L3\ (Q\text{-trapping})}$ shifts the ion trapping location more towards Q2. This causes the ions to gain more v_z as VDC_{Q2} is higher than VDC_{Q3} and results in the ion beam to strike more towards +z direction (Figure S23g). In contrast, the potential gradient between the LQ-trap and L3 during the ion ejection reduces when the $V_{L3\ (Ejection\ to\ pusher)}$ is higher, which causes the ions to travel slowly. These slow moving ion cloud appear more to the right of the pusher compared to the position where it appear when subjected to low $V_{L3\ (Ejection\ to\ pusher)}$, prior to the pulsing of the ions to the orthogonal TPX3 detector. Thus an increase in $V_{L3\ (Ejection\ to\ pusher)}$ causes the ion beam to strikes more to the –z direction of the orthogonal detector (Figure S23h).

Parameter	Value
**Orbitrap MS parameters**	
Mas resolution (@200 *m/z*)	70,000
Polarity	Positive
Average	0
Microscans	1
nESI source spray voltage (kV)	1.2
Capillary temperature (°C)	320
In-source trapping desolvation voltage (V)	-50
Noise threshold	4.64
Inject time (ms)	90
Ion tranfer *m/z* optimization	High *m/z*
Detector *m/z* optimization	Low *m/z*
Trapping gas pressure setting	3
Extended trapping (eV)	3
**Orbitrap settings for external instrument mode of operation (External Orbitrap, axial non-imaging and orthogonal TOF non-imaging modes)**	
HCD external instrument mode	On
HCD offset to external instrument (V)	35
HCD offset from external instrument (V)	2
HCD gradient to external instrument (V)	-200
HCD gradient from external instrument (V)	45
HCD and C-trap exit lens close (V)	35
HCD exit lens open to external instrument (V)	25
HCD exit lens open from external instrument (V)	-25
HCD ion tranfer (ms)	25
HCD waiting for external instrument back transfer (ms)	250
HCD exit lens trigger voltage low (V)	35
HCD exit lens trigger voltage high (V)	-35
**TOF analyzer & associated tranfer ion optics settings**	
LQ-trap pressure (mbar)	1.00E-04
TOF analyzer pressure (mbar)	9.00E-07
RF voltage of the hexapole and LQ-trap (V)	350
C1 (1st electrode of correction lens) voltage (V)	16 (Axial non-imaging and orthogonal TOF non-imaging modes)
Voltages on the 4 segments (top, bottom, left, right) of C2 (2nd electrode of correction lens) (V)	-7, -7, -4, -4 (Axial non-imaging and orthogonal TOF non-imaging modes)
C3 (3rd electrode of correction lens) (V)	-15 (Axial non-imaging and orthogonal TOF non-imaging modes)
Lens 4 voltage (V)	-35 (Axial non-imaging and orthogonal TOF non-imaging modes)
Vs (V)	3333 (Orthogonal TOF non-imaging mode)
Vd (V)	6666 (Orthogonal TOF non-imaging mode)
Axial detector voltage (V)	-1800
Orthogonal detector voltage (V)	-2800
Oscilloscope sampling rate (ns)	0.5

Table S1. Data acquisition parameters corresponds to Figure S13.

Parameter	Value
**Orbitrap MS parameters**	
Mas resolution (@200 *m/z*)	70,000
Polarity	Positive
Average	10
Microscans	1
nESI source spray voltage (kV)	1.2
Capillary temperature (°C)	320
In-source trapping desolvation voltage (V)	-100
Noise threshold	3.64
Inject time (ms)	90
Ion tranfer *m/z* optimization	Low *m/z*
Detector *m/z* optimization	Low *m/z*
Trapping gas pressure setting	1
Extended trapping (eV)	3
**Orbitrap settings for external instrument mode of operation (External Orbitrap mode)**	
HCD external instrument mode	On
HCD offset to external instrument (V)	35
HCD offset from external instrument (V)	2
HCD gradient to external instrument (V)	-200
HCD gradient from external instrument (V)	45
HCD and C-trap exit lens close (V)	35
HCD exit lens open to external instrument (V)	25
HCD exit lens open from external instrument (V)	-25
HCD ion tranfer (ms)	25
HCD waiting for external instrument back transfer (ms)	250
HCD exit lens trigger voltage low (V)	35
HCD exit lens trigger voltage high (V)	-35
**TOF analyzer & associated tranfer ion optics settings**	
LQ-trap pressure (mbar)	5.00E-05
TOF analyzer pressure (mbar)	6.00E-07
RF voltage of the hexapole and LQ-trap (V)	200

Table S2. Parameters used for the acquisition of data corresponds to Figure S19-21.

Parameter	Value
**Orbitrap MS parameters**	
Mas resolution (@200 *m/z*)	4,375
Polarity	Positive
Average (Data aquistion)	0
Microscans	1
Number of scans averaged	3
nESI source spray voltage (kV)	1.2
Capillary temperature (°C)	320
In-source trapping desolvation voltage (V)	-50
Noise threshold	4.64
Inject time (ms)	90
Ion tranfer *m/z* optimization	High *m/z*
Detector *m/z* optimization	Low *m/z*
Trapping gas pressure setting	5
Extended trapping (eV)	3
**Orbitrap settings for external instrument mode of operation**	
HCD external instrument mode	On
HCD offset to external instrument (V)	35
HCD offset from external instrument (V)	2
HCD gradient to external instrument (V)	-200
HCD gradient from external instrument (V)	45
HCD and C-trap exit lens close (V)	35
HCD exit lens open to external instrument (V)	25
HCD exit lens open from external instrument (V)	-25
HCD ion tranfer (ms)	25
HCD waiting for external instrument back transfer (ms)	250
HCD exit lens trigger voltage low (V)	35
HCD exit lens trigger voltage high (V)	-35
**TOF analyzer & associated tranfer ion optics settings**	
LQ-trap pressure (mbar)	3.40E-04
TOF analyzer pressure (mbar)	1.20E-06
RF voltage of the hexapole and LQ-trap (V)	600
Lens 3 voltage (V)	35
Laser pulse energy (mJ)	1.5 to 2.25
Atttenuator knob position & laser pulse exit energy (mJ)	680 & 0.43 to 0.65

Table S3. Parameters used for the acquisition of data corresponds to Figure 4a.

Parameter	Value
**Orbitrap MS parameters**	
Polarity	Positive
nESI source spray voltage (kV)	1.4
Capillary temperature (°C)	320
In-source trapping desolvation voltage (V)	-50
Inject time (ms)	90
Ion tranfer *m/z* optimization	High *m/z*
Detector *m/z* optimization	Low *m/z*
Trapping gas pressure setting	3
Extended trapping (eV)	3
**Orbitrap settings for external instrument mode of operation**	
HCD external instrument mode	On
HCD offset to external instrument (V)	35
HCD offset from external instrument (V)	2
HCD gradient to external instrument (V)	-200
HCD gradient from external instrument (V)	45
HCD and C-trap exit lens close (V)	35
HCD exit lens open to external instrument (V)	25
HCD exit lens open from external instrument (V)	-25
HCD ion tranfer (ms)	25
HCD waiting for external instrument back transfer (ms)	250
HCD exit lens trigger voltage low (V)	35
HCD exit lens trigger voltage high (V)	-35
**TOF analyzer & associated tranfer ion optics settings**	
LQ-trap pressure (mbar)	8.50E-05
TOF analyzer pressure (mbar)	7.00E-07
RF voltage of the hexapole and LQ-trap (V)	400
C1 (1st electrode of correction lens) voltage (V)	11
Voltages on the 4 segments (top, bottom, left, right) of C2 (2nd electrode of correction lens) (V)	0, -2, -0, 0
C3 (3rd electrode of correction lens) (V)	-20
Lens 4 voltage (V)	-35
Vs (V)	3333
Vd (V)	6666
Laser pulse energy (mJ)	1.75
Atttenuator knob position & laser pulse exit energy (mJ)	680 & 0.5
Orthogonal MCP front plate voltage (V)	-2200
Orthogonal MCP back plate voltage (V)	-600
Phosphor screen voltage (V)	5000
TPX3CAM f-stop value	0.95

Table S4. Parameters used for the acquisition of data corresponds to Figure 5.

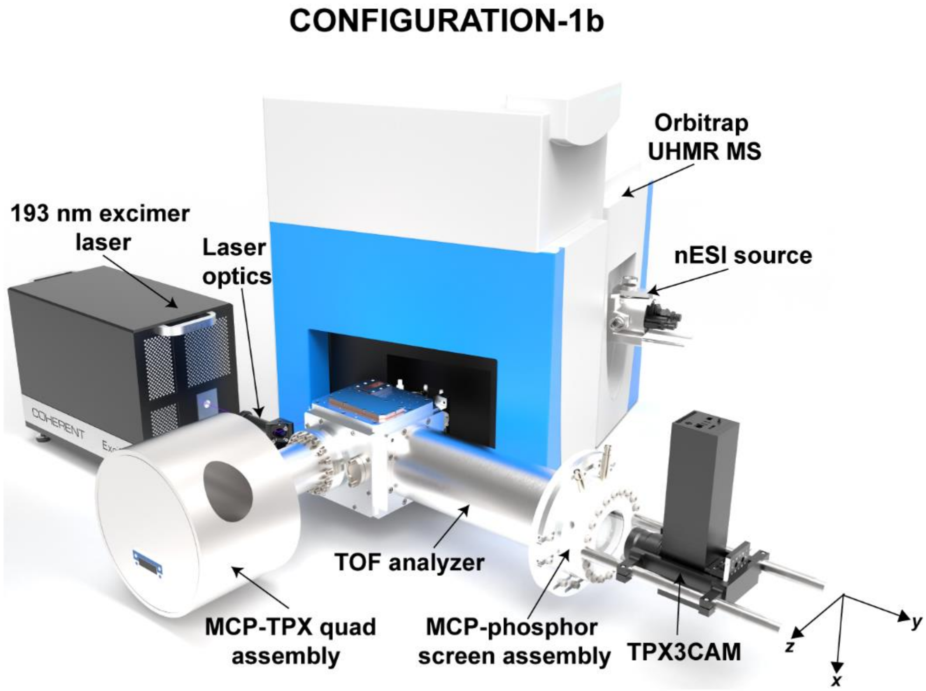

Figure S1. (a) Schematic (not to scale) of the Orbitrap/TOF instrument with axial MCP-TPX quad and orthogonal MCP-phosphor screen (P47)-TPX3CAM imaging detector assemblies, and orthogonal 193 nm excimer laser optics (System configuration 1b).

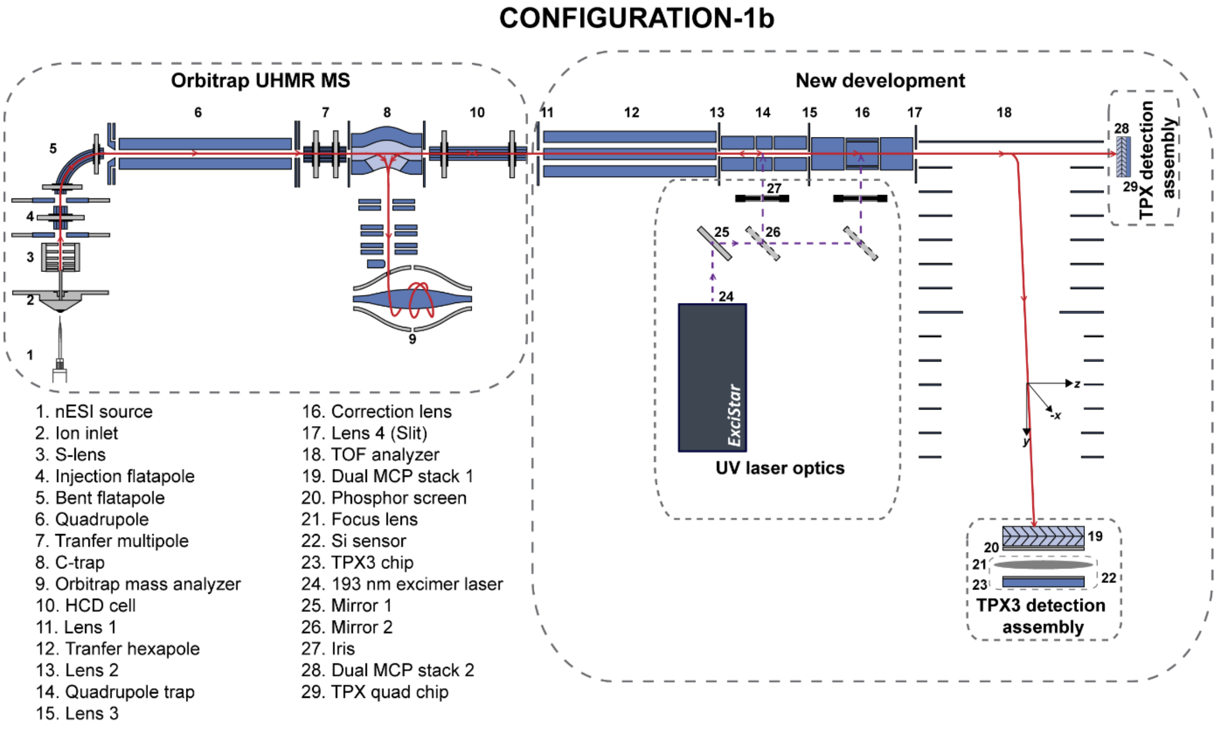

Figure S2. Detailed ion and laser optics schematic (not to scale) of configuration 1b of the Orbitrap/TOF system.

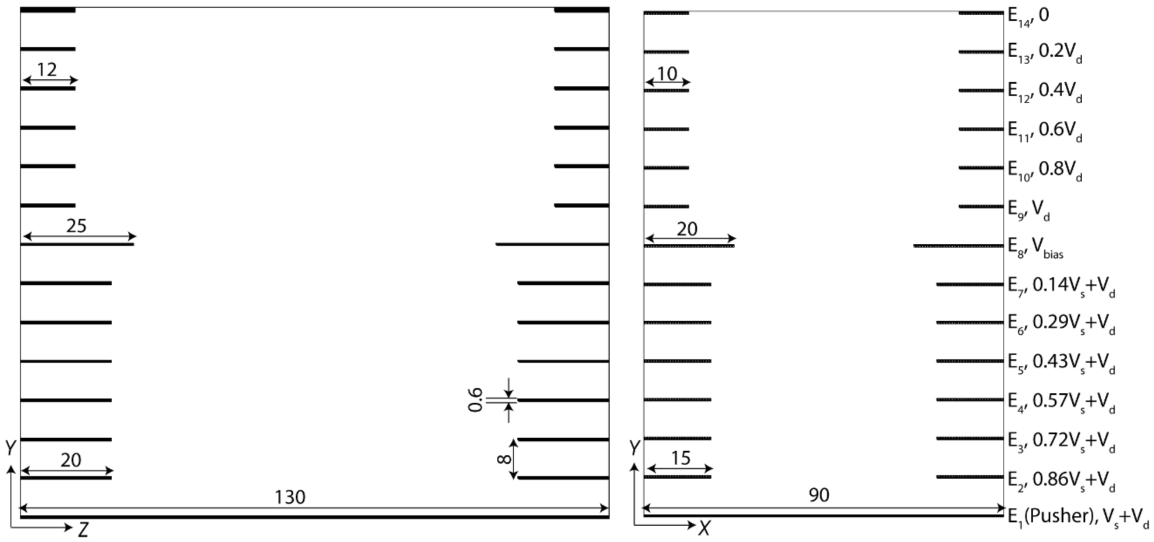

Figure S3. Ion optics schematic of the custom designed TOF analyzer. TOF analyzer consists of 14 electrodes with two stage acceleration field (s and d fields) and a bias electrode. The dimensions (in mm) and voltage division between the electrodes are shown. Distance from the pusher (E_1) to the orthogonal detector=655 mm.

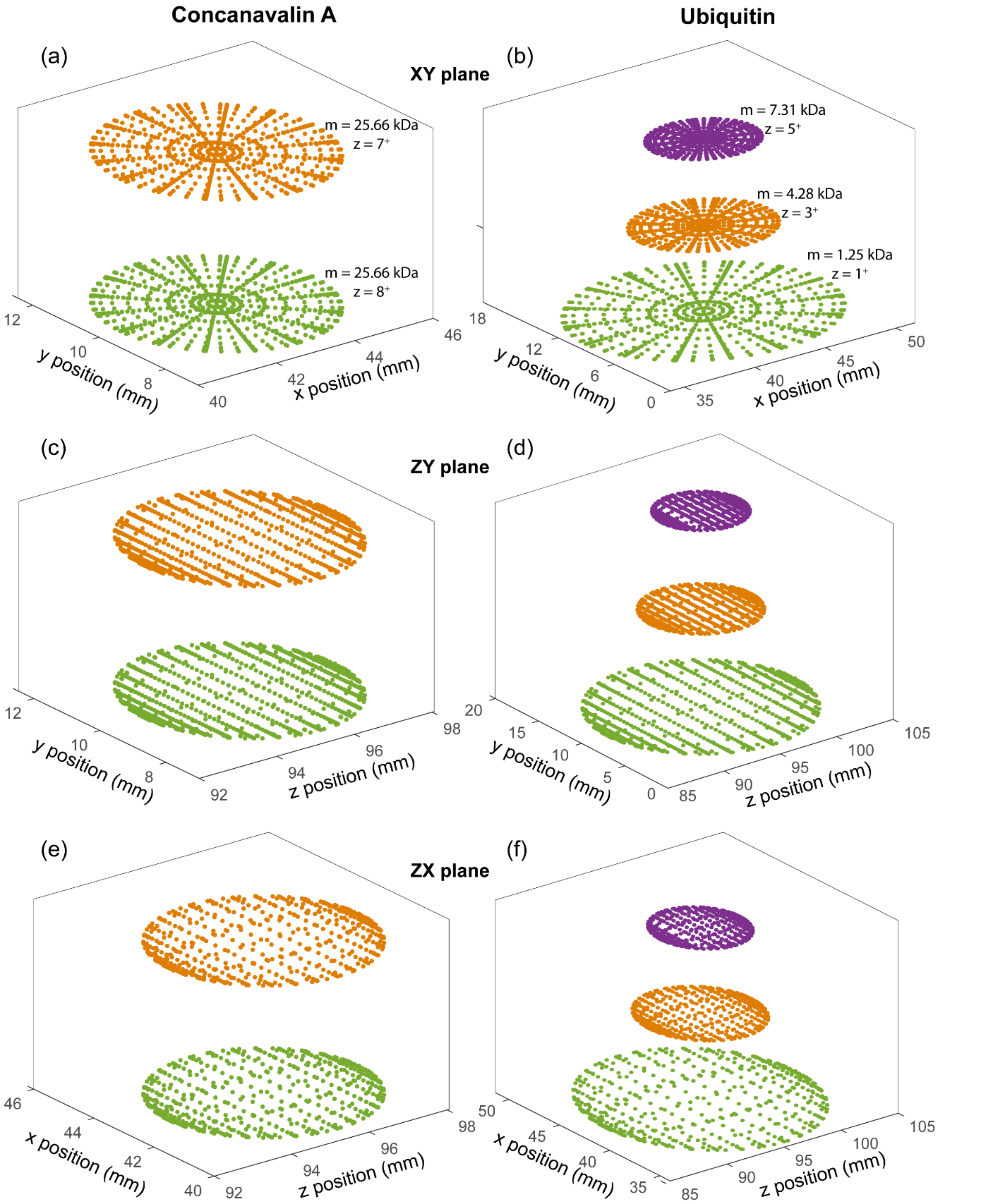

Figure S4. Projections of the simulated isotropic distribution of the fragments formed from concanavalin A and ubiquitin in XY (a and b), ZY (c and d) and ZX (e and f) plane in the field free region after 20 µs of the dissociation. Note that $E_{Fragmentation}$ of 2 eV/fragment was considered.

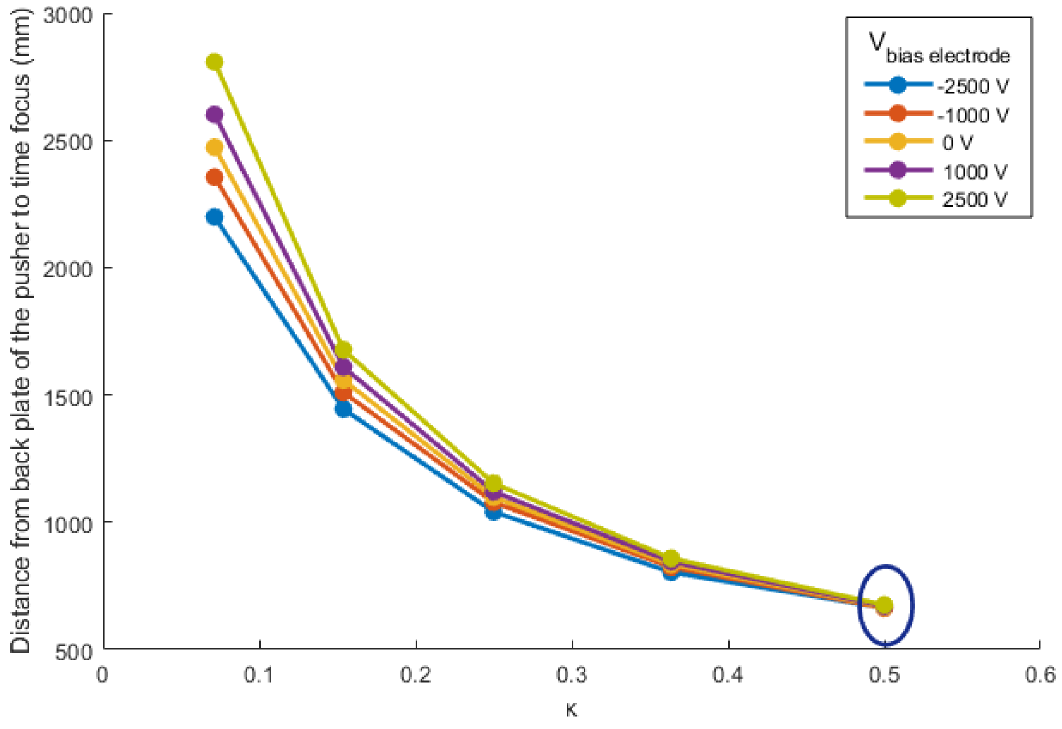

Figure S5. Simulation results showing the effect of V_s, V_d and $V_{bias\ electrode}$ on the time focus at a nominal orthogonal acceleration potential of 15 kV (pusher voltage). The time focus value displays in the y-axis is calculated by measuring the distance from the pusher to the TOF region with the minimum spatial spread of the ion beam in y (orthogonal)-direction. κ is the ratio of V_s to V_d. 8+ charged concanavalin A monomer fragment ion cloud with the following properties was used for the determination of time focus; E_{Axial}=225 eV, $E_{Fragmentation}$=2 eV, Fragmentation location: midpoint of the pusher.

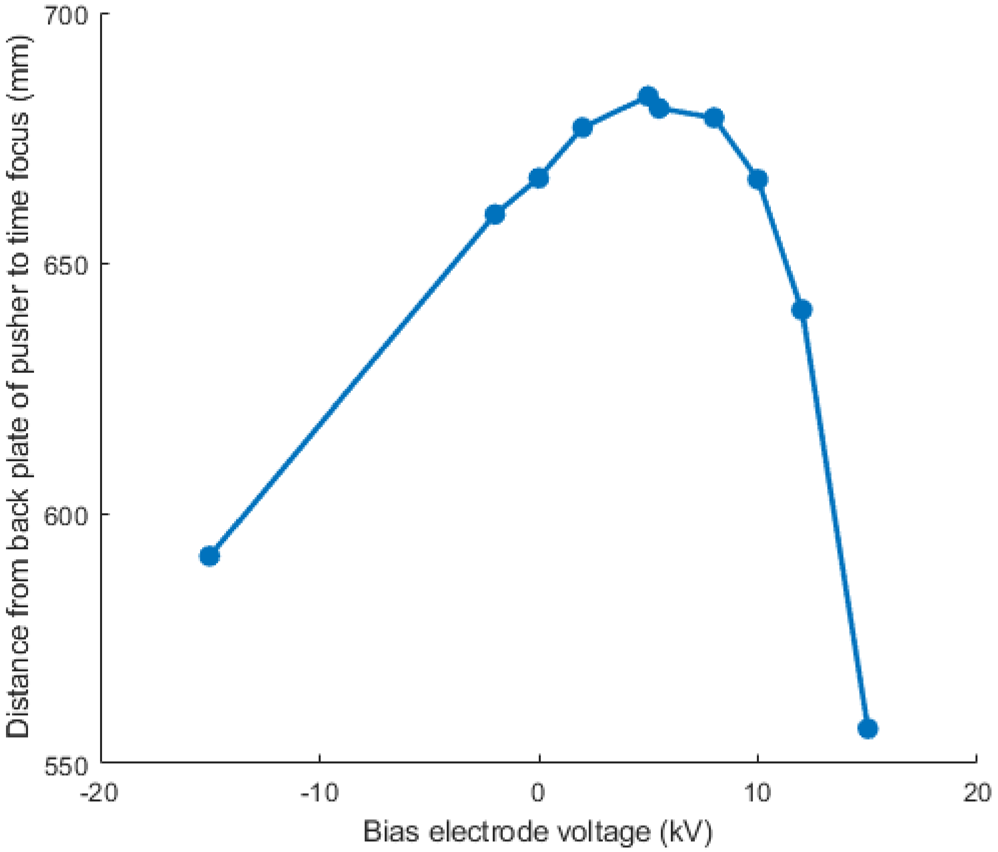

Figure S6. Simulation results showing the effect of $V_{bias\ electrode}$ on the time focus at κ=0.5 (V_s=5 kV and V_d=10 kV). The time focus drifts by a maximum distance of 130 mm for a change in $V_{bias\ electrode}$ from -15 to +15 kV. 8+ charged concanavalin A monomer fragment ion cloud with the following properties was used for the determination of time focus; E_{Axial}=225 eV, $E_{Fragmentation}$=2 eV, Fragmentation location: midpoint of the pusher.

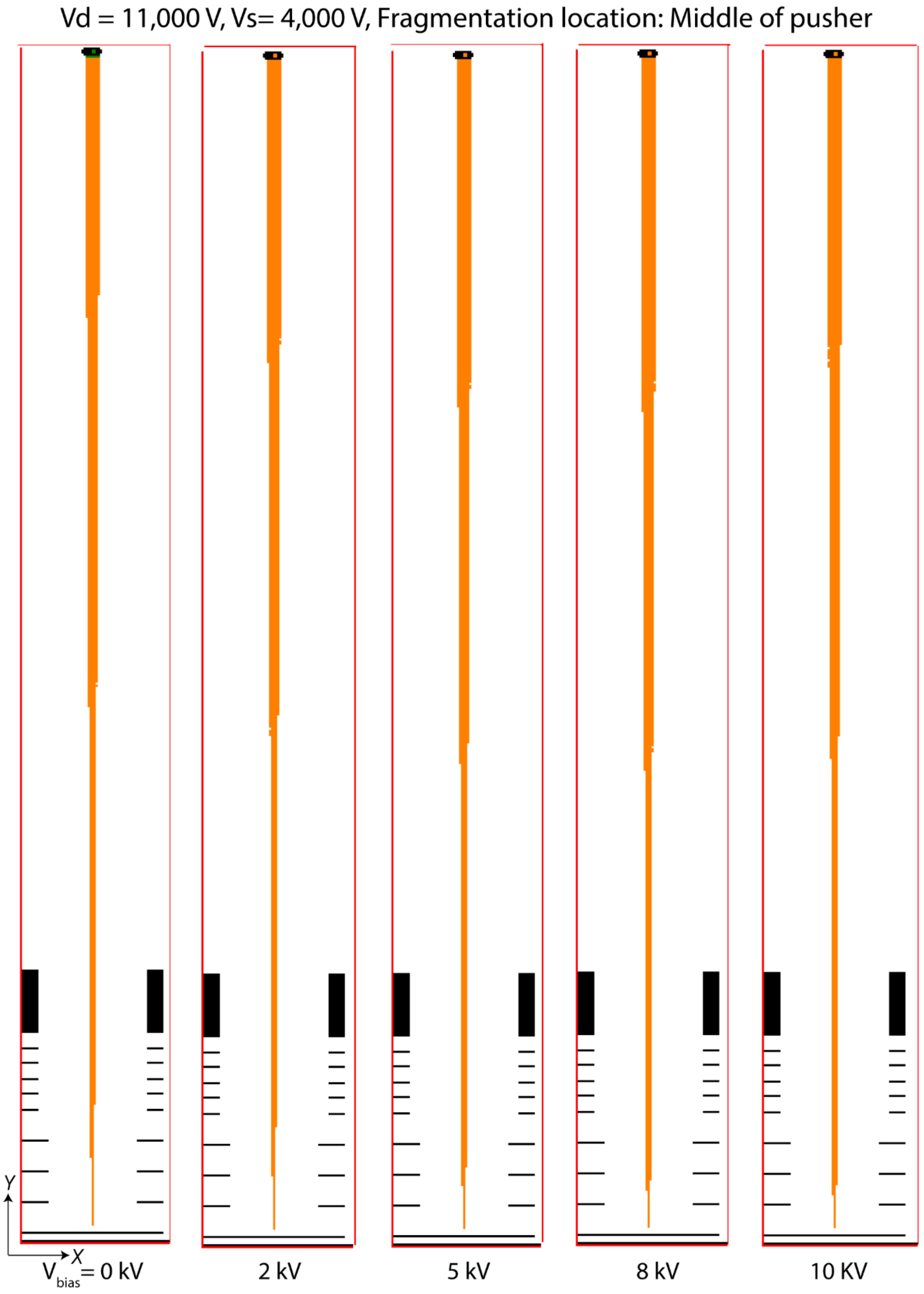

Figure S7. Simulated ion trajectories (in xy plane) of the concanavalin A $[M+8H]^{8+}$ and $[M+7H]^{7+}$ monomer fragment ion clouds, correspond to different $V_{bias\ electrode}$ at κ = 0.364 (V_s=4000 V, V_d=11,000 V). E_{Axial}=225 eV, $E_{Fragmentation}$=2 eV, Fragmentation location: midpoint of the pusher.

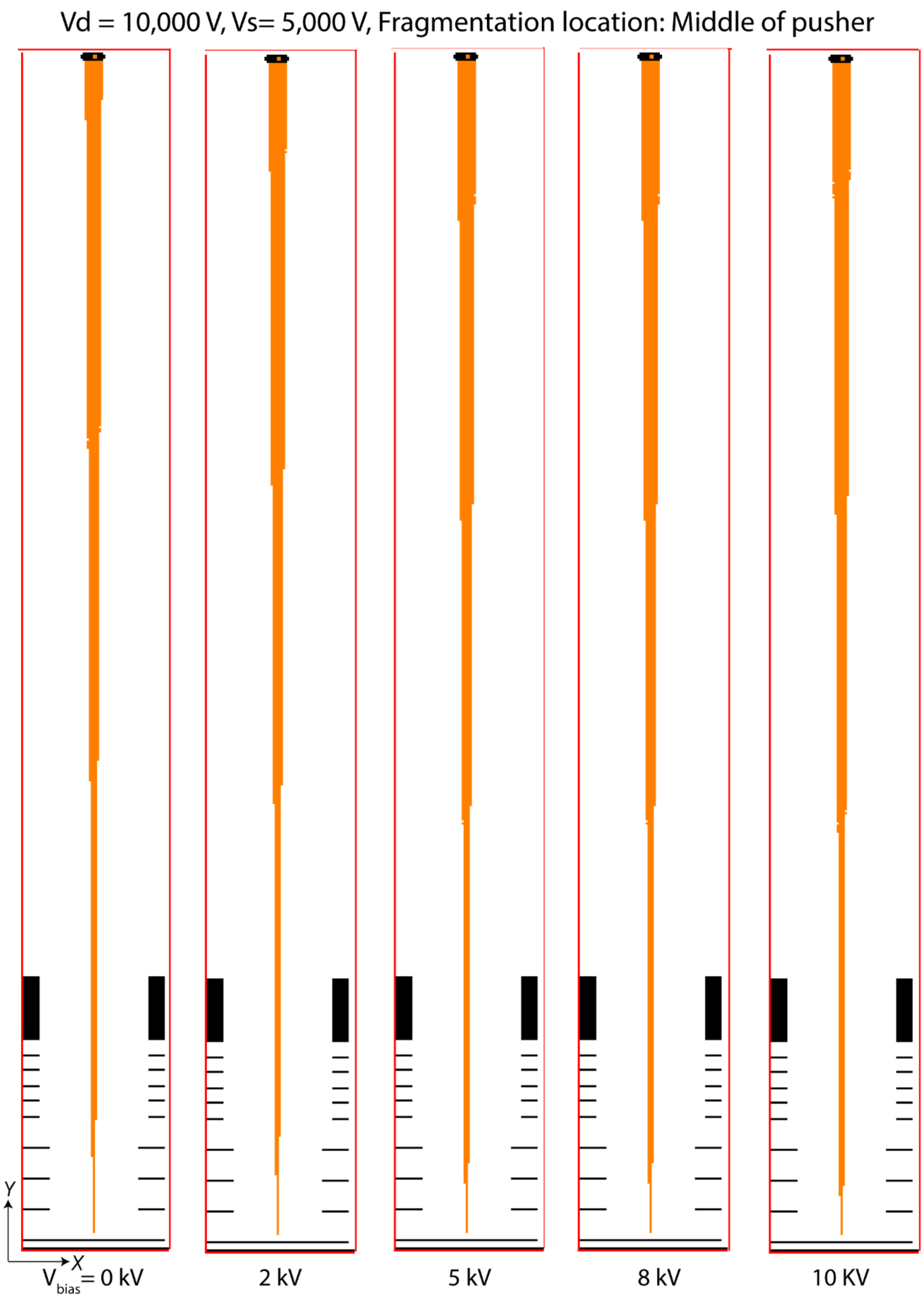

Figure S8. Simulated ion trajectories (in xy plane) of the concanavalin A $[M+8H]^{8+}$ and $[M+7H]^{7+}$ monomer fragment ion clouds, correspond to different $V_{bias\ electrode}$ at κ = 0.5 (V_s=5000 V, V_d=10,000 V). E_{Axial}=225 eV, $E_{Fragmentation}$=2 eV, Fragmentation location: midpoint of the pusher.

Vd = 11,000 V, Vs= 4,000 V, Fragmentation location: Middle of C2

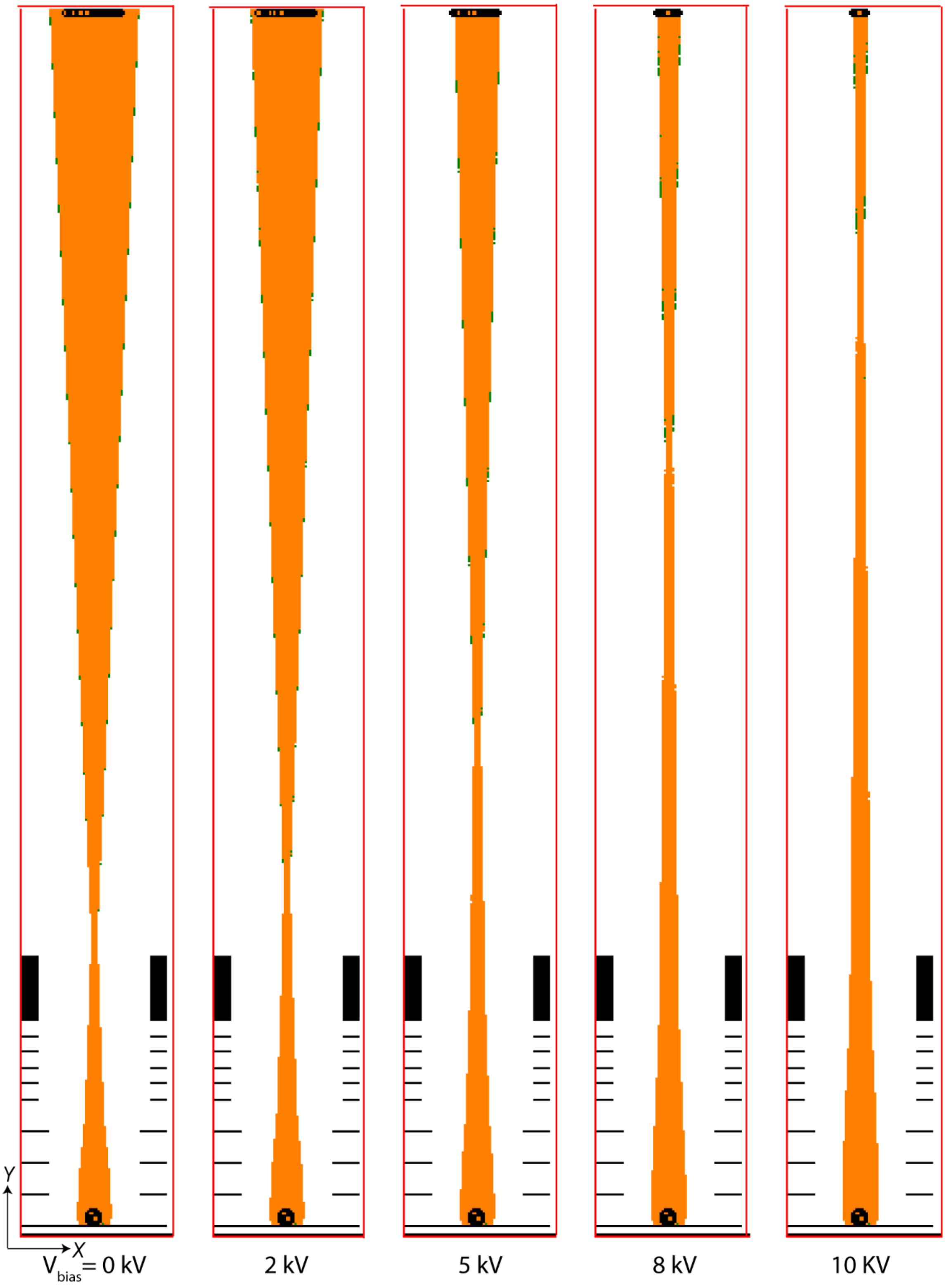

Figure S9. Simulated ion trajectories (in xy plane) of the concanavalin A $[M+8H]^{8+}$ and $[M+7H]^{7+}$ monomer fragment ion clouds, correspond to different $V_{bias\ electrode}$ at κ = 0.364 (V_s=4000 V, V_d=11,000 V). E_{Axial}=225 eV, $E_{Fragmentation}$=2 eV, Fragmentation location: middle of the second segment of the correction lens (C2).

Vd = 10,000 V, Vs= 5,000 V, Fragmentation location: Middle of C2

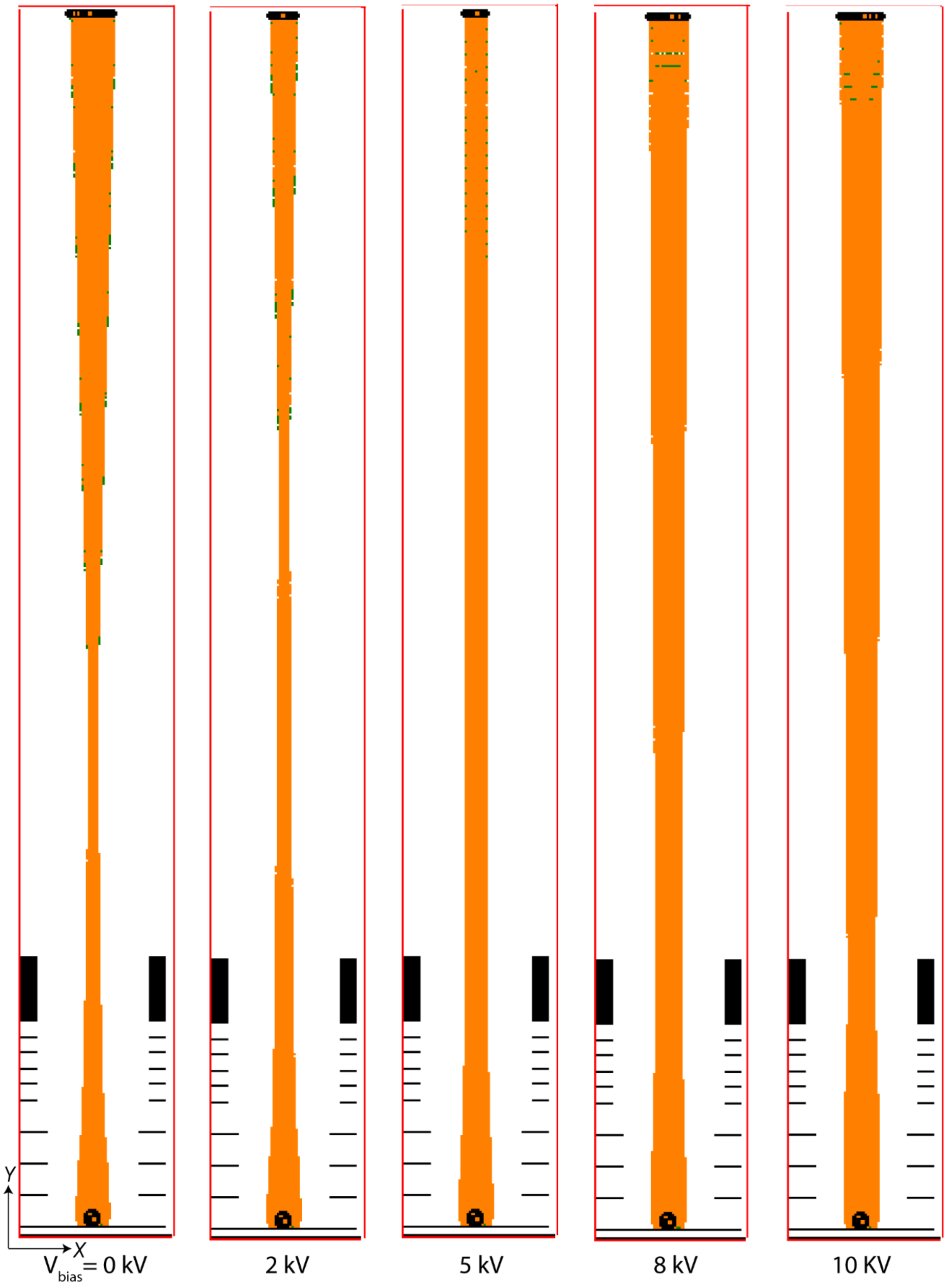

Figure S10. Simulated ion trajectories (in xy plane) of the concanavalin A $[M+8H]^{8+}$ and $[M+7H]^{7+}$ monomer fragment ion clouds, correspond to different $V_{bias\ electrode}$ at κ = 0.5 (V_s=5000 V, V_d=10,000 V). E_{Axial}=225 eV, $E_{Fragmentation}$=2 eV, Fragmentation location: middle of the second segment of the correction lens (C2).

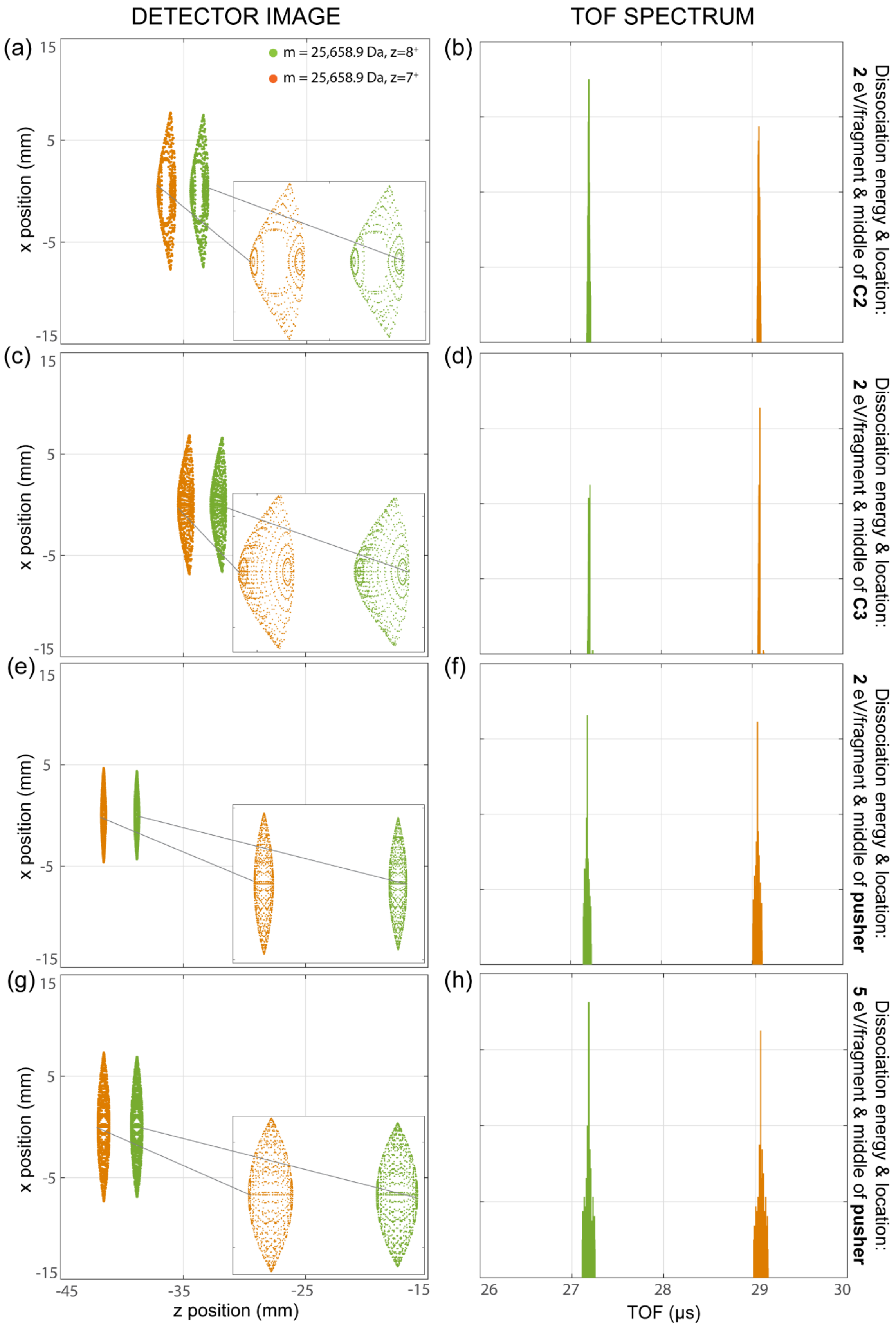

Figure S11. Simulated detector images (a, c, e and g) and TOF spectra (b, d, f and h) collected at the orthogonal detector at different dissociation energies (2-5 eV/fragment) and locations (C2, C3 and pusher) by sending concanvalin A monomer $[M+8H]^{8+}$ (green) and $[M+7H]^{7+}$(orange) fragment ions with an axial energy of 225 eV per fragment.

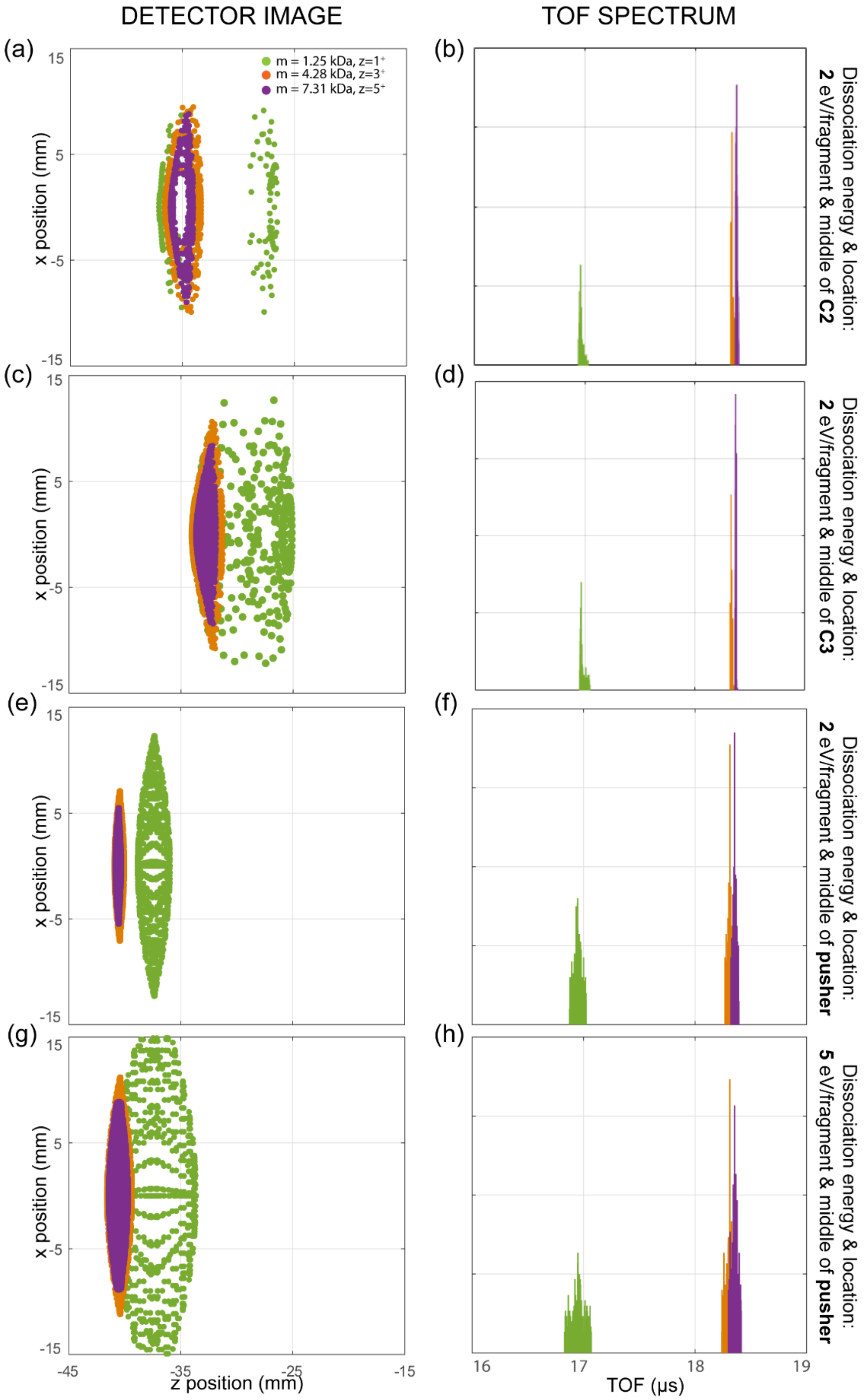

Figure S12. Simulated detector images (a, c, e and g) and TOF spectra (b, d, f and h) collected at the orthogonal detector at different dissociation energies (2-5 eV/fragment) and locations (C2, C3 and pusher) by sending ubiquitin fragments of *m/z*=1247 (green), 1428 (orange) and 1463 (purple) fragment ions. Precursor ubiquitin ion was accelerated with an axial voltage of 30 V.

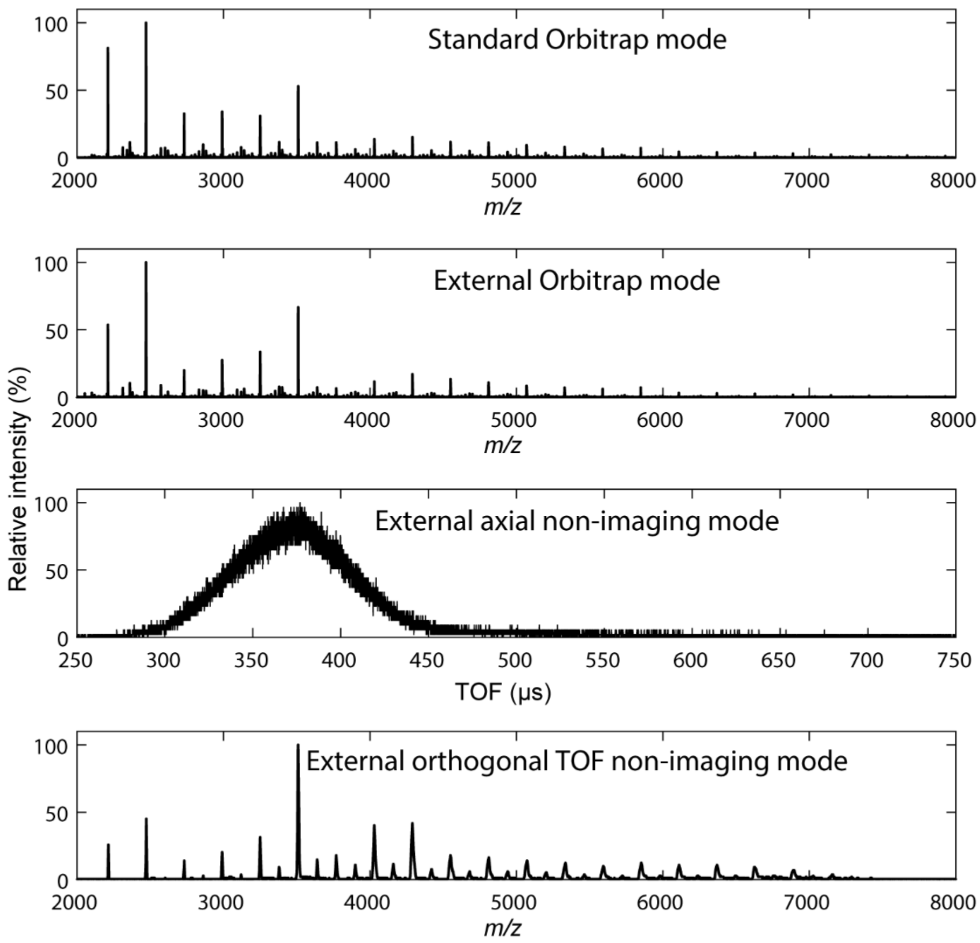

Figure S13. Mass spectra of CsI mix acquired by operating the Orbitrap/TOF instrument in different modes using Orbitrap and discrete-dynode EM detectors (non-imaging) under identical ion optical conditions. Table S1 and Figures S14-18 show the relevant data acquisition parameters and event sequences used in different operation modes.

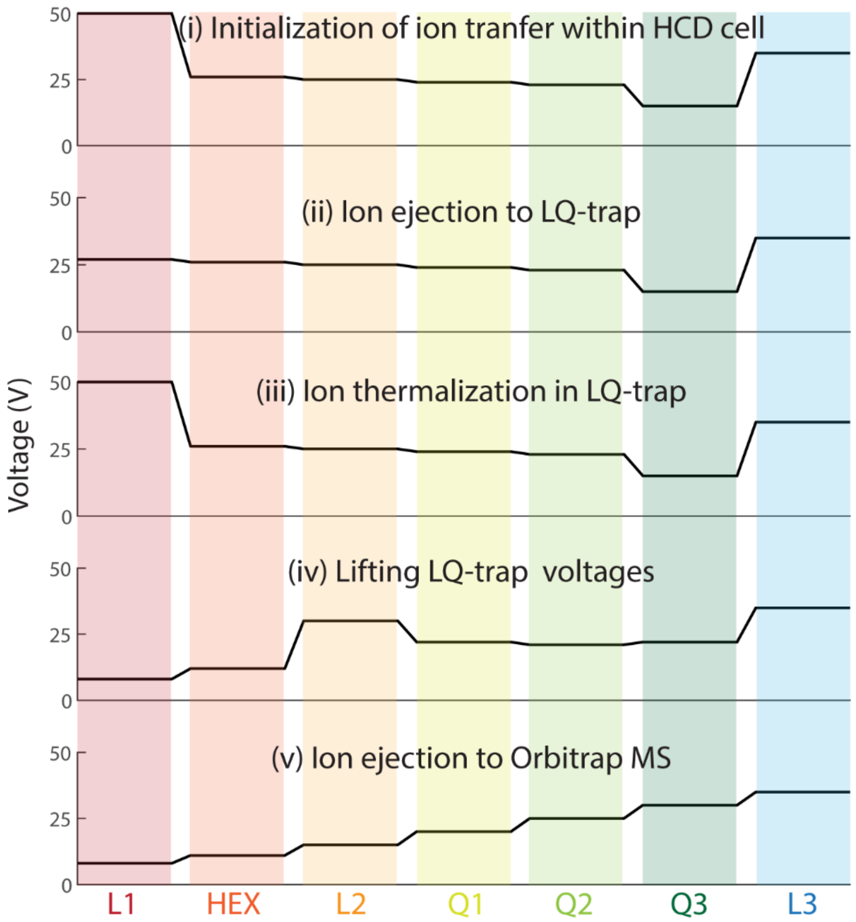

Figure S14. Transitions of the DC voltage profile across the ion optics during each event used for the acquisition of the CsI mix (Figure S13) and concanavalin A (Figure 4a) spectra in external Orbitrap mode (L1-3: Lens 1-3, HEX: Hexapole, Q1-3: Quadrupole electrode 1-3).

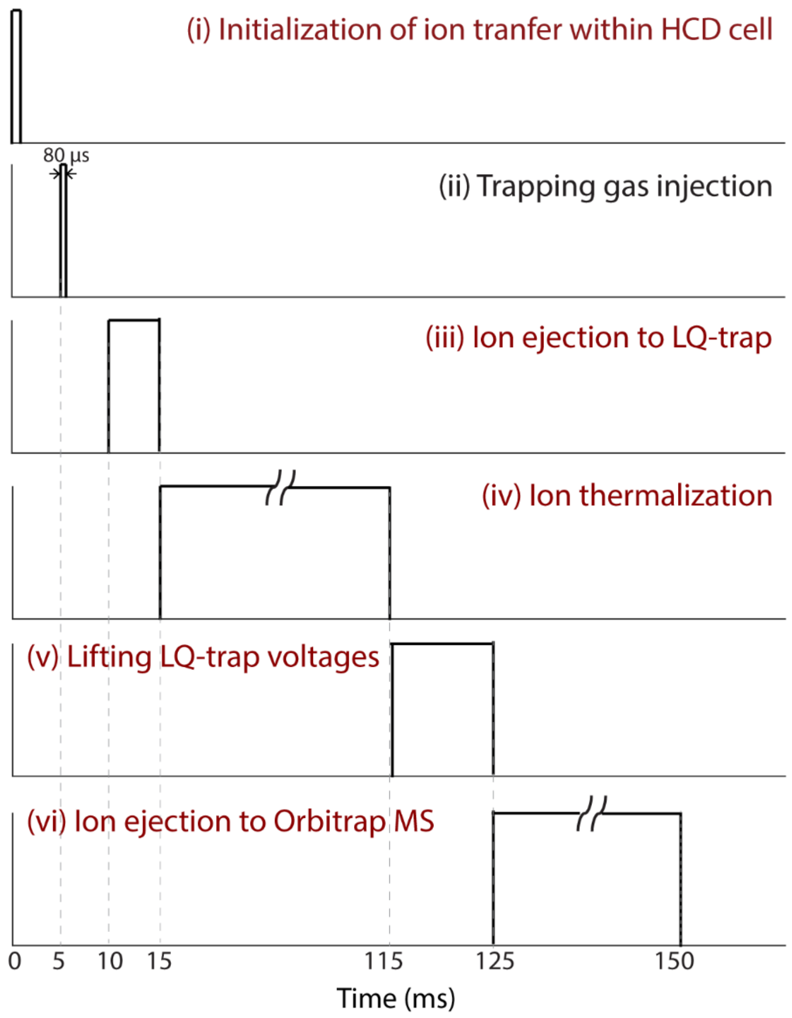

Figure S15. Timing diagram of the events used for the acquisition of the CsI mix (Figure S13) and ubiquitin (Figures S19-21) spectra in external Orbitrap mode. Note that the timing signals represented in red (title) are associated with a change in DC voltage profile of the ion optics elements 11-15 (Figures 2, S14 (CsI mix) and S22 (Ubiquitin)).

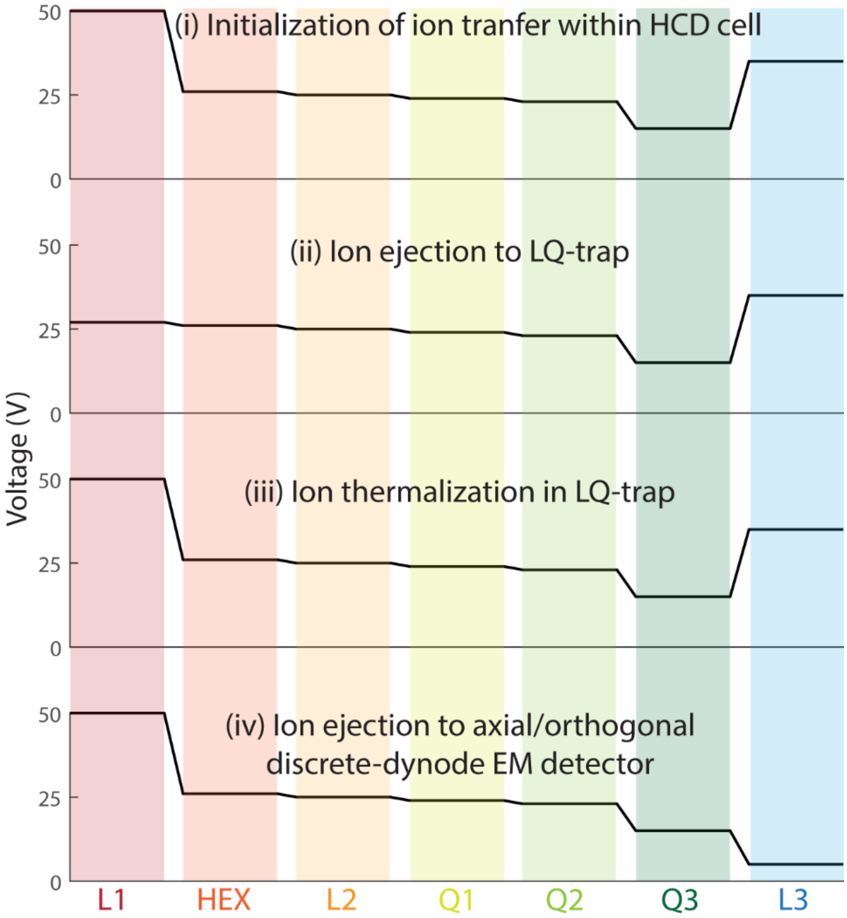

Figure S16. Transitions of the DC voltage profile across the ion optics during each event used for the acquisition of the CsI mix spectrum in external axial non-imaging and orthogonal TOF non-imaging modes (Figure S13) and concanavalin A UVPD spectrum in external UVPD orthogonal TOF imaging mode (Figure 5) (L1-3: Lens 1-3, HEX: Hexapole, Q1-3: Quadrupole electrode 1-3).

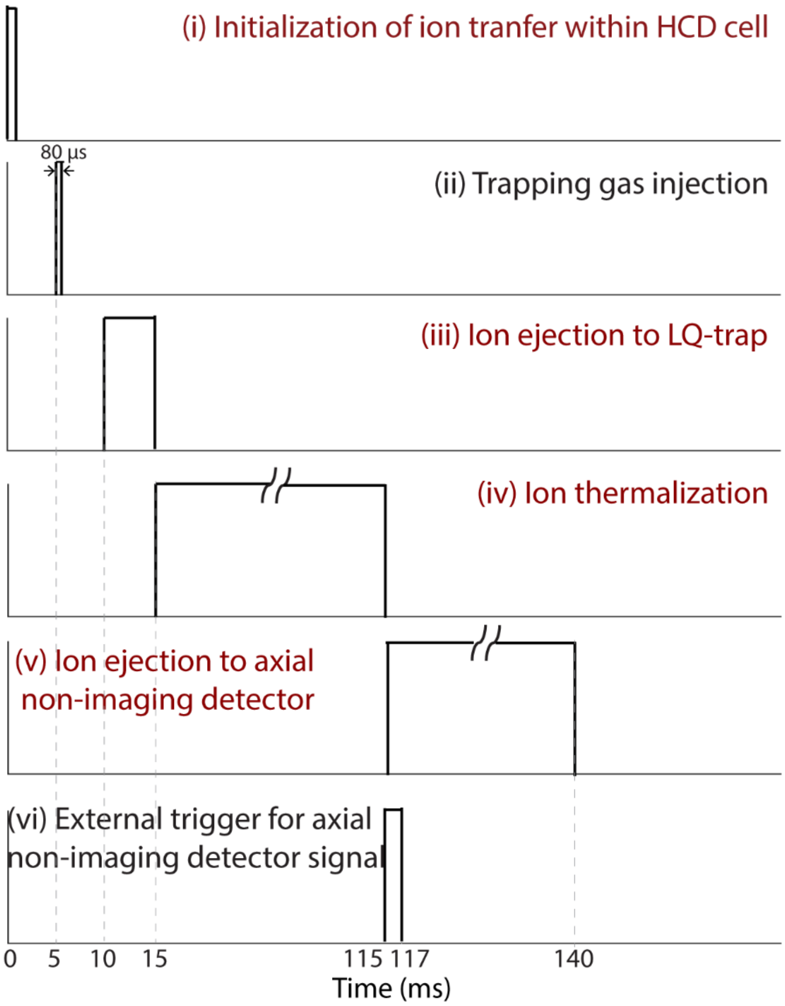

Figure S17. Timing diagram of the events used for the acquisition of the CsI mix spectrum in external axial non-imaging mode (Figure S13). Note that the timing signals represented in red (title) are associated with a change in the voltage profile of ion optics elements 11-15 (Figures 2 and S16 (CsI mix)).

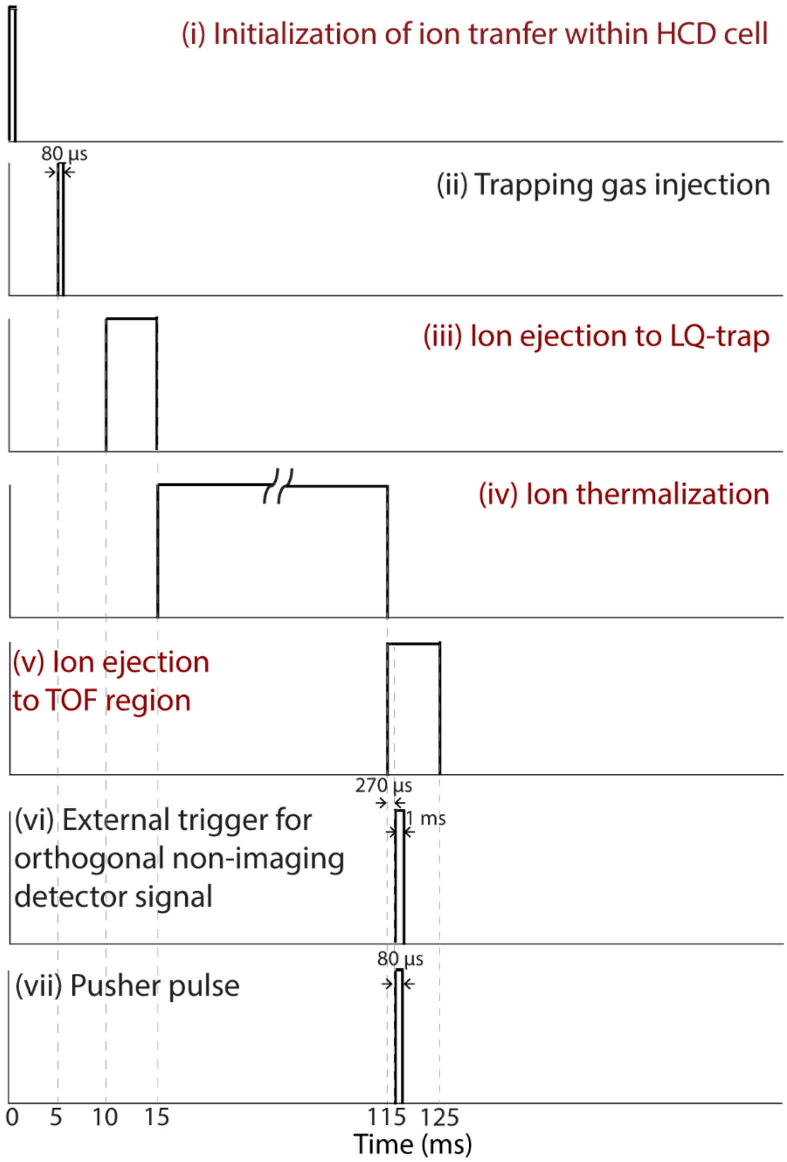

Figure S18. Timing diagram of the events used for the acquisition of CsI mix spectrum in external orthogonal TOF non-imaging mode (Figure S13). Note that the timing signals represented in red (title) are associated with a change in the voltage profile of ion optics elements 11-15 (Figure 2, S16 (CsI mix)).

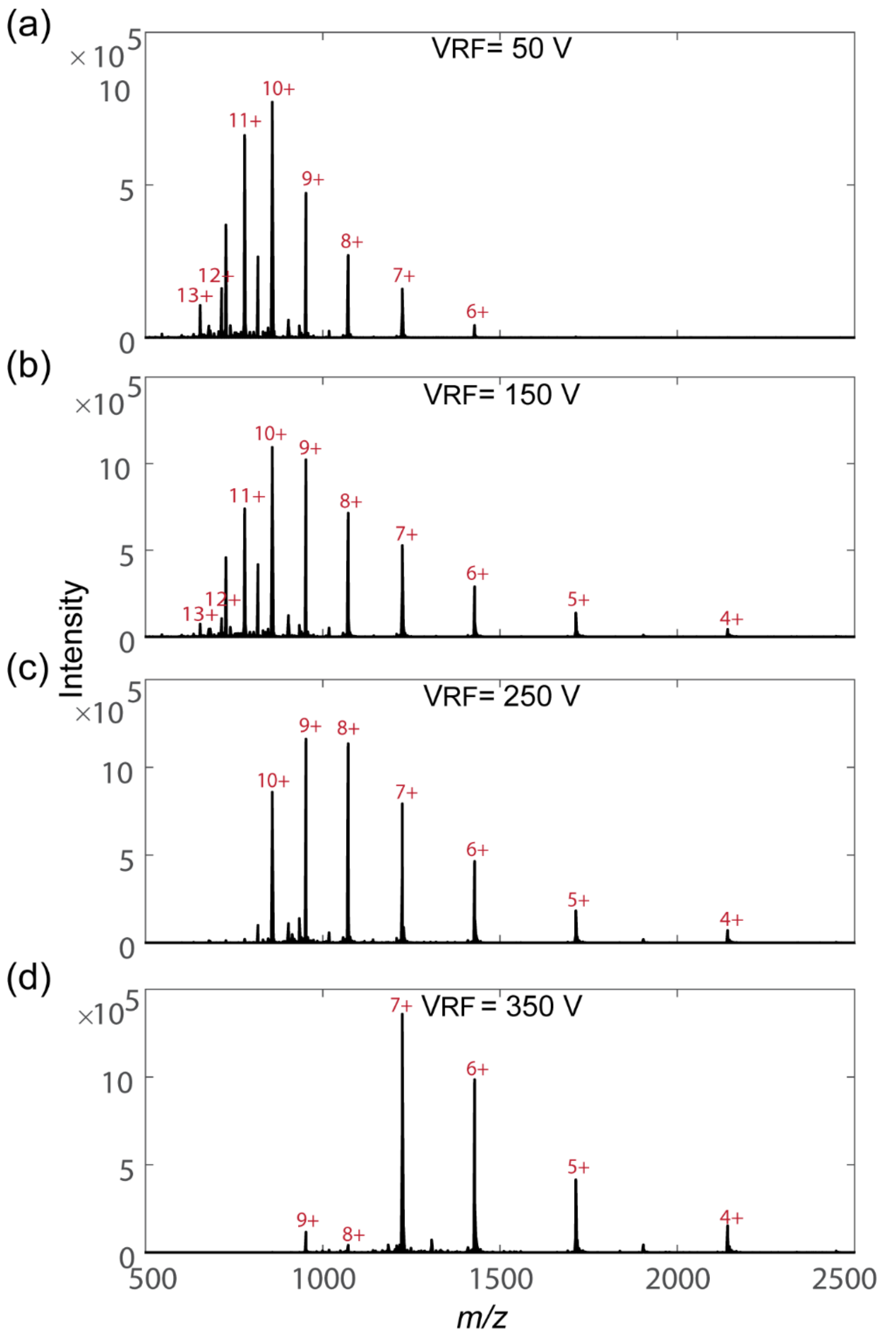

Figure S19. (a-d) Denatured ubiquitin spectra measured in external Orbitrap mode at different RF voltages of the hexapole and LQ-trap (VRF).

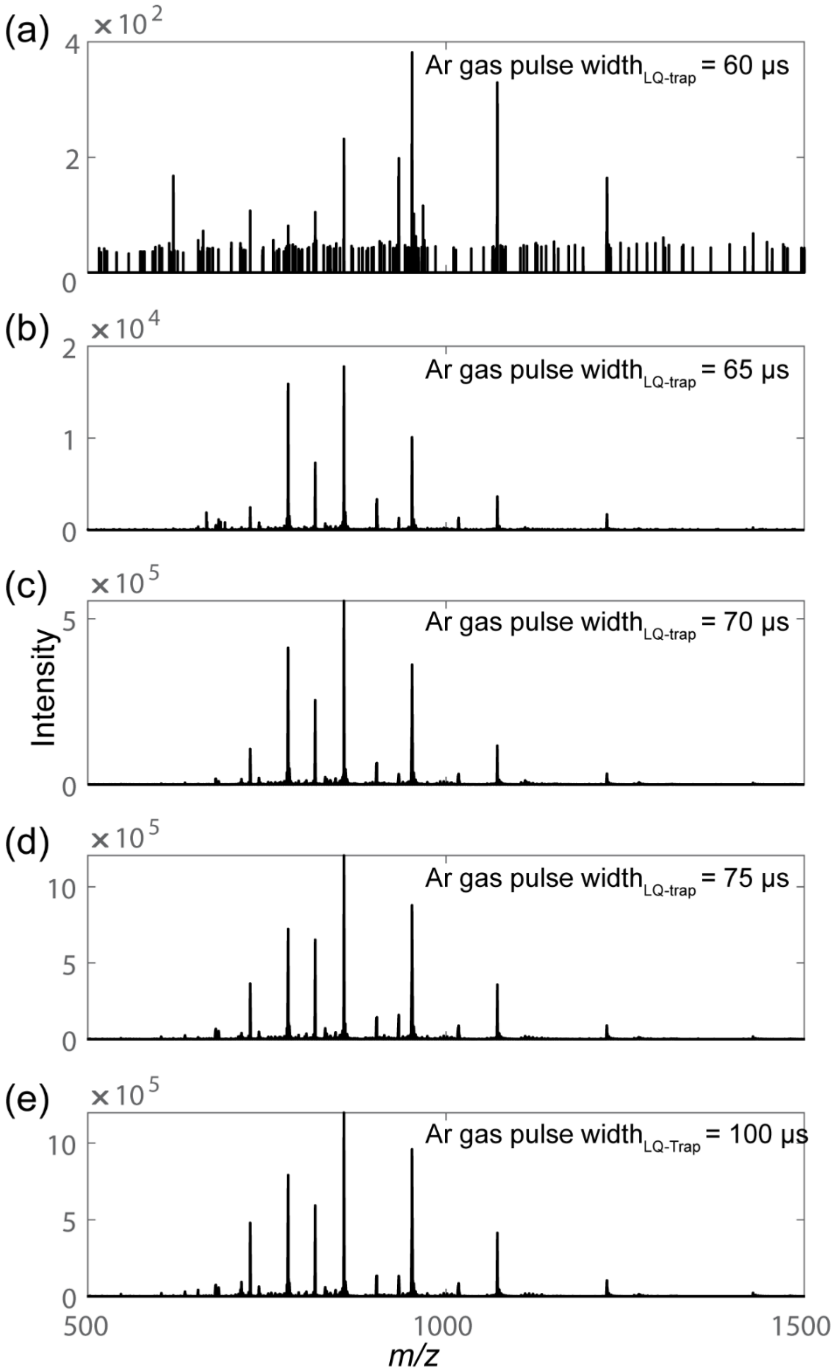

Figure S20. (a-e) Denatured ubiquitin spectrum measured in external Orbitrap mode at different trapping gas (Ar) gas pulse widths.

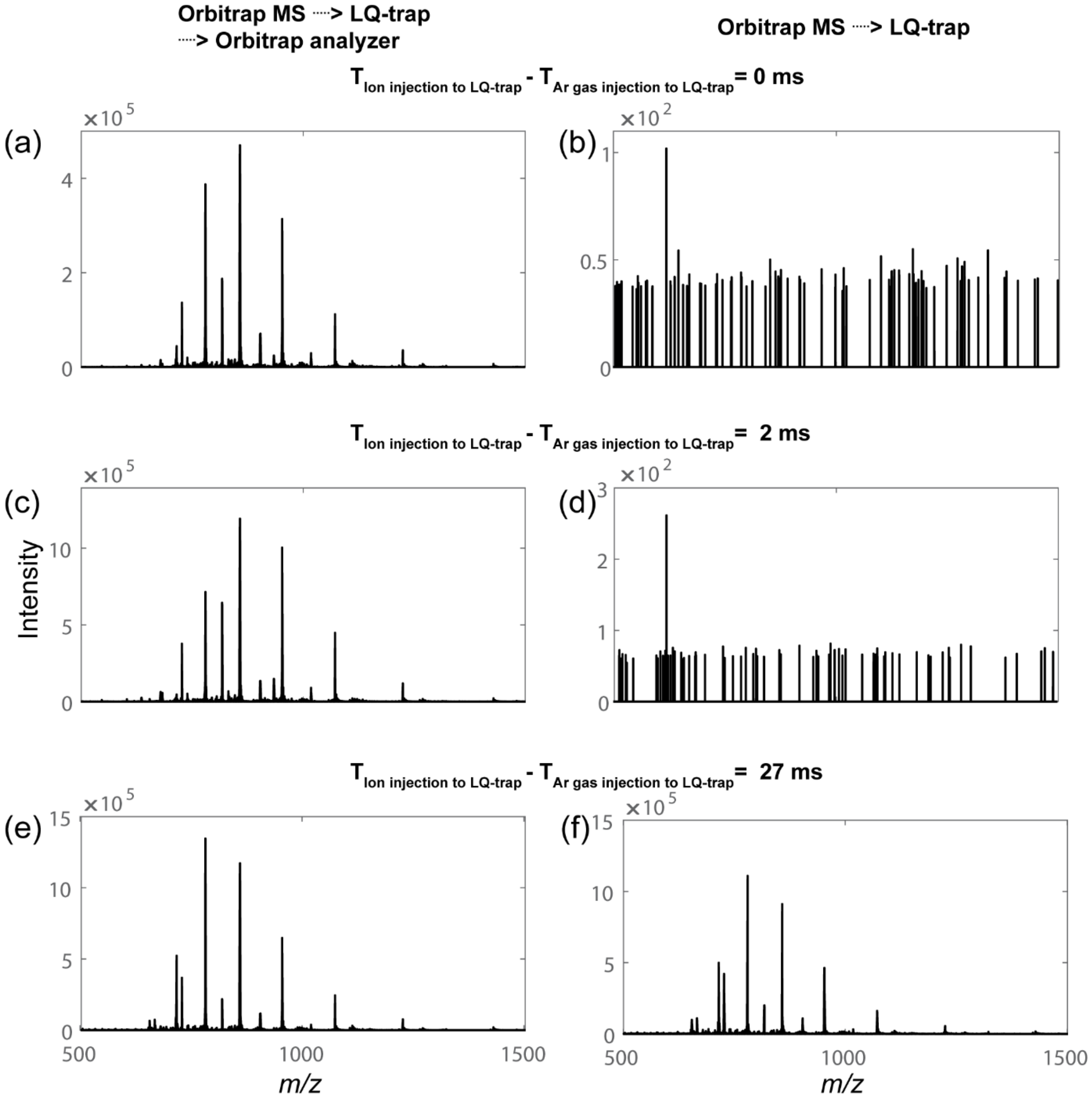

Figure S21. Influence of $T_{\text{Ar gas injection to LQ-trap - Ion injection to LQ-trap}}$ on the Orbitrap spectrum of denatured ubiquitin. All of the left sided spectra (a, c and e) were recorded in the external Orbitrap mode. All of the right sided Orbitrap spectra (b, d and f) were collected by sending the ions from the HCD cell to the LQ-trap and storing them there instead of sending back to the Orbitrap MS by applying a high voltage of 50 V to lens 1.

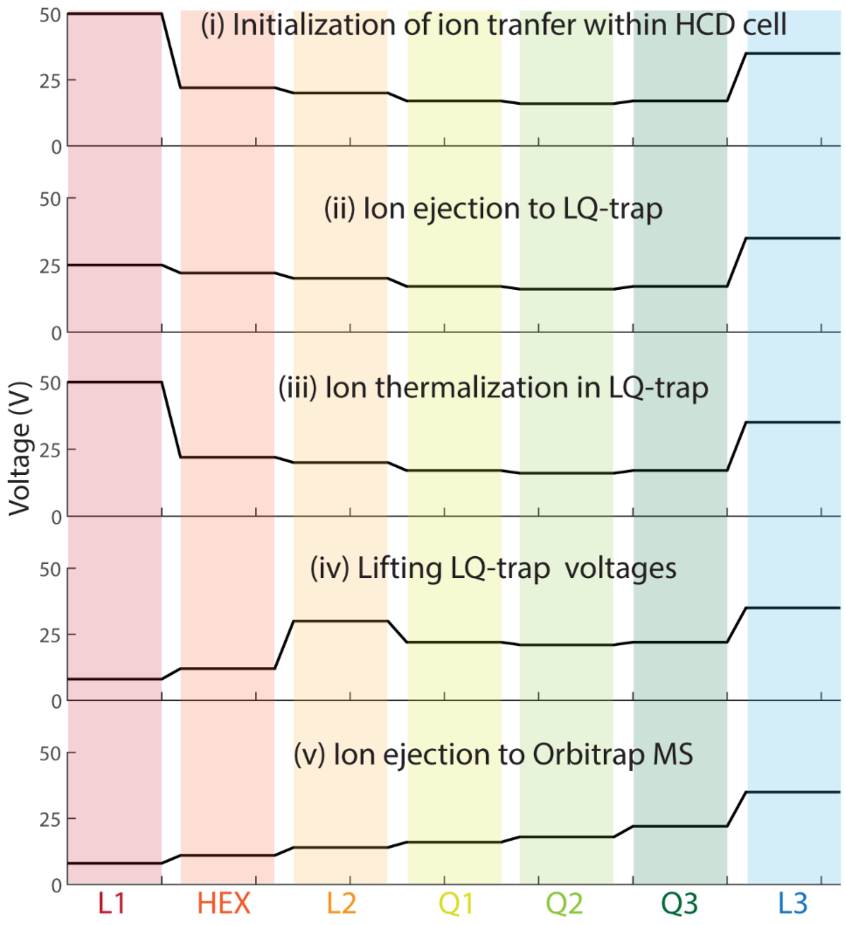

Figure S22. Transitions of the DC voltage profile across the ion optics during each event used for the acquisition of the ubiquitin spectrum in external Orbitrap mode (Figure S19-21) (L1-3: Lens 1-3, HEX: Hexapole, Q1-3: Quadrupole segment 1-3).

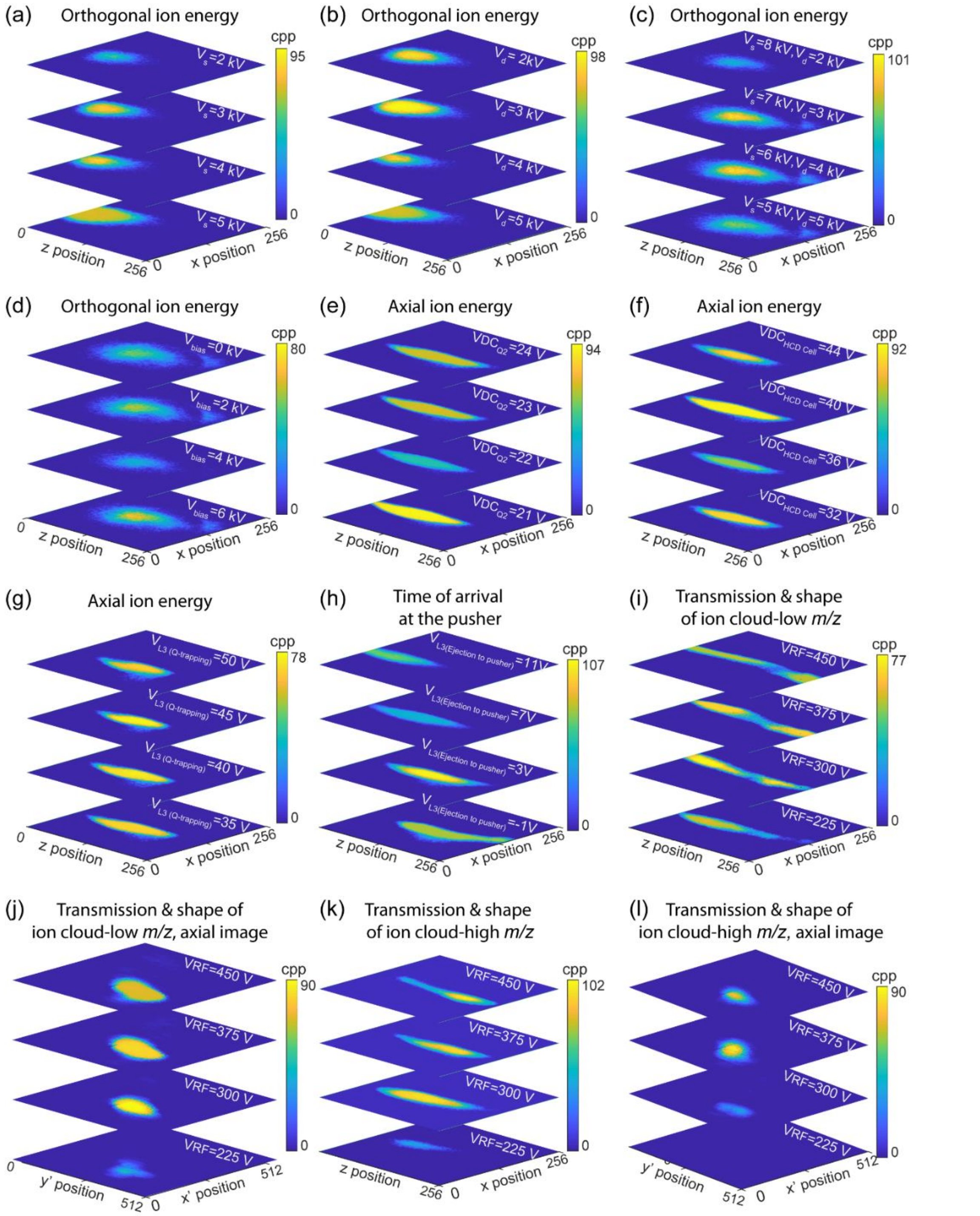

Figure S23. Influence of (a) V_s (V_d=5 kV), (b) V_d (V_s=5 kV), (c) s & d fields (V_s+V_d=10 kV), (d) V_{bias} (V_s=5 kV, V_d=5 kV), (e) voltage on the second segment of the LQ-trap (VDC_{Q2}),(f) DC offset voltage of the HCD cell ($VDC_{HCD\ cell}$), (g) lens 3 voltage during the ion storage in LQ-trap ($V_{L3\ (Q\text{-}trapping)}$) and (h) lens 3 voltage during the ejection from the LQ-trap to TOF region ($V_{L3\ (Ejection\ to\ pusher)}$) on the spatial distribution of quadrupole selected CsI singly charged ions with *m/z*=5589 at the orthogonal TPX3 detector. Spatial profiles of the quadrupole isolated ions with *m/z* values of 3510.5 (i and j) and 7407.7 (k and l) at the orthogonal TPX3 and axial TPX quad detectors at different RF voltages of the hexapole and LQ-trap (VRF) values. All images represents the sum of hundred measurement cycles. cpp = counts per pixel.

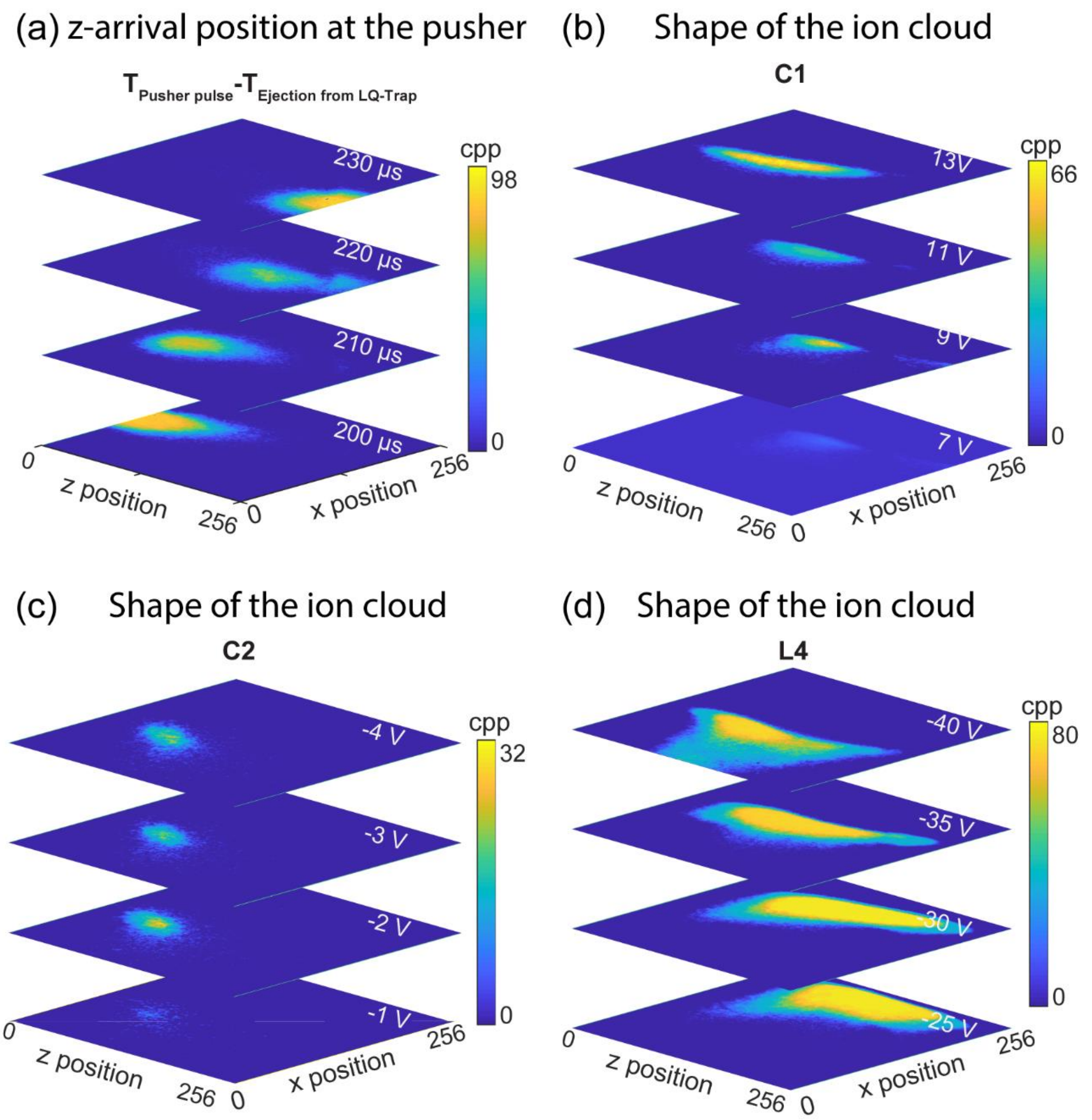

Figure S24. Influence of (a) $T_{\text{Pusher pulse}}$-$T_{\text{Ejection from LQ-trap}}$ (time difference between the ion ejection from the LQ-trap and pusher pulsing), voltage on the first (b, C1) and second (c, C2) electrode of the correction lens and (d) lens 4 (L4) voltage on the spatial distribution of quadrupole selected CsI singly charged ions with *m/z*=5589 at the orthogonal TPX3 detector. All images represents the sum of hundred measurement cycles. cpp = counts per pixel.

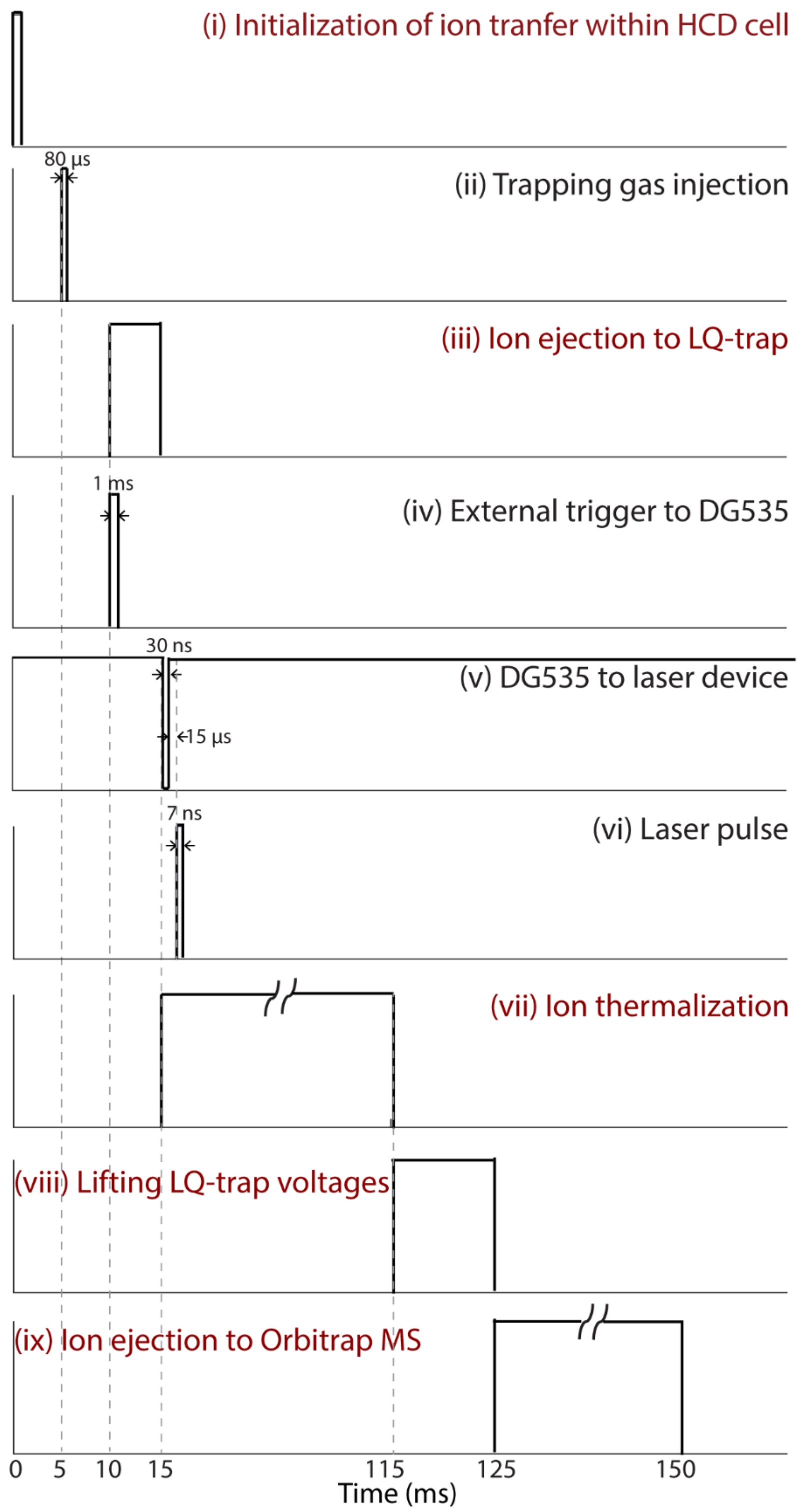

Figure S25. Timing diagram of the events used for the acquisition of concanavalin A UVPD spectrum in external UVPD Orbitrap mode (Figure 4a). Note that the timing signals represented in red (title) are associated with a change in the voltage profile of ion optics elements 11-15 (Figures 2 and S14 (Concanavalin A)).

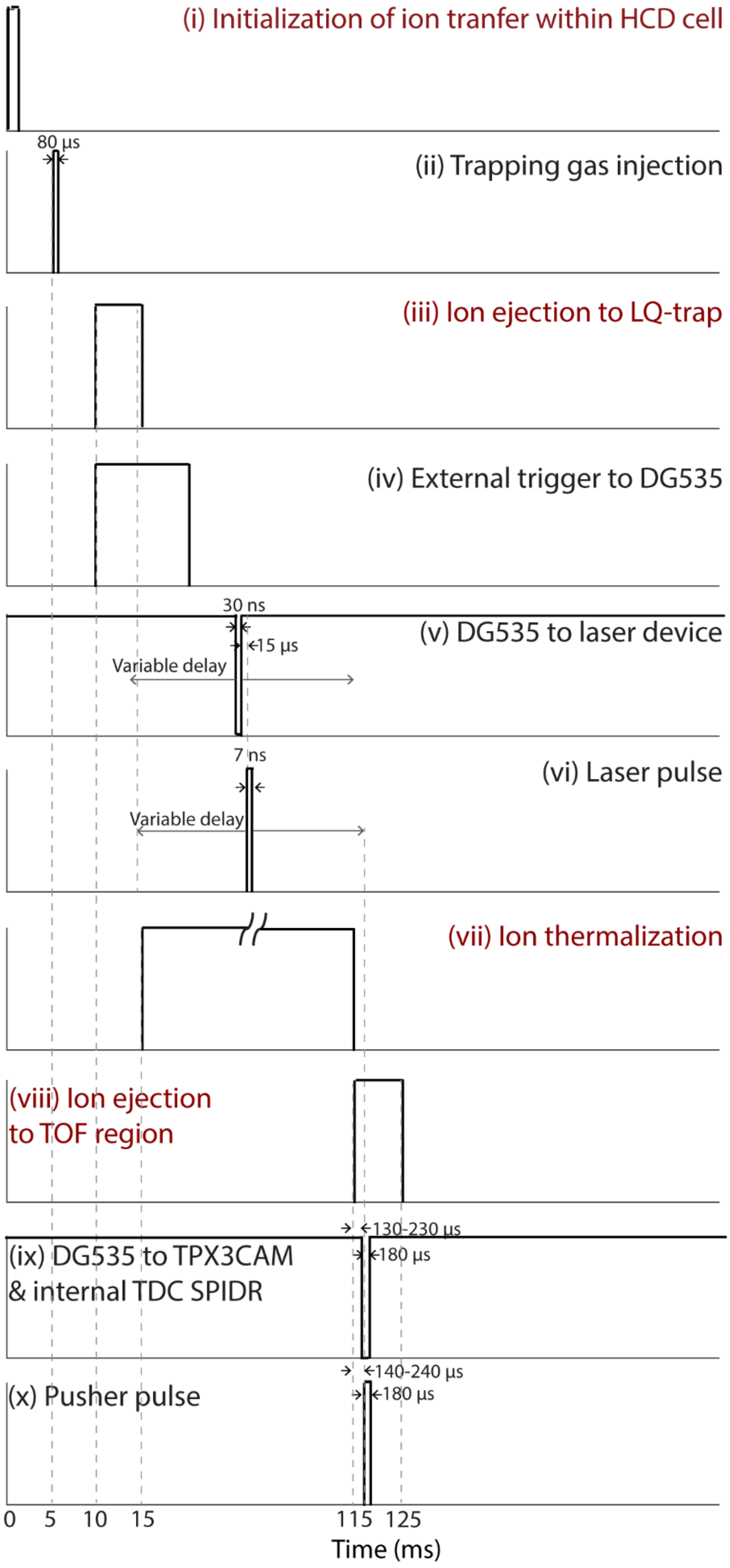

Figure S26. Timing diagram of the events used for the acquisition of concanavalin A UVPD spectrum in external UVPD orthogonal TOF imaging mode (Figure 5). Note that the timing signals represented in red (title) are associated with a change in the voltage profile of ion optics elements 11-15 (Figure 2 and S16 (concanavalin A)).

References

1. Kelly, R. T.; Tolmachev, A. V.; Page, J. S.; Tang, K.; Smith, R. D., The ion funnel: theory, implementations, and applications. *Mass Spectrometry Reviews* **2010,** *29* (2), 294-312.
2. Leiminger, M.; Feil, S.; Mutschlechner, P.; Ylisirniö, A.; Gunsch, D.; Fischer, L.; Jordan, A.; Schobesberger, S.; Hansel, A.; Steiner, G., Characterisation of the transfer of cluster ions through an atmospheric pressure interface time-of-flight mass spectrometer with hexapole ion guides. *Atmospheric Measurement Techniques* **2019,** *12* (10), 5231-5246.
